# Early autonomous patterning of the anteroposterior axis in gastruloids

**DOI:** 10.1242/dev.202171

**Published:** 2024-11-18

**Authors:** Kerim Anlaş, Nicola Gritti, Fumio Nakaki, Laura Salamó Palau, Sham Leilah Tlili, David Oriola, Krisztina Arató, Jia Le Lim, James Sharpe, Vikas Trivedi

**Affiliations:** ^1^ EMBL Barcelona, 08003 Barcelona, Spain; ^2^ Aix-Marseille Univ., CNRS, UMR 7288, IBDM, Turing Center for Living Systems, 13288 Marseille, France; ^3^ Institució Catalana de Recerca i Estudis Avançats, 08010 Barcelona, Spain; ^4^ EMBL Heidelberg, Developmental Biology Unit, 69117 Heidelberg, Germany

**Keywords:** Anteroposterior axis, Symmetry breaking, Embryonic patterning, Gastruloids, Stem cell aggregates, Cell states

## Abstract

Minimal *in vitro* systems composed of embryonic stem cells (ESCs) have been shown to recapitulate the establishment of the anteroposterior (AP) axis. In contrast to the native embryo, ESC aggregates – such as gastruloids – can break symmetry, which is demarcated by polarization of the mesodermal marker *T*, autonomously without any localized external cues. However, associated earliest patterning events, such as the spatial restriction of cell fates and concomitant transcriptional changes, remain poorly understood. Here, we dissect the dynamics of AP axis establishment in mouse gastruloids, particularly before external *Wnt* stimulation. Through single-cell RNA sequencing, we identify key cell state transitions and the molecular signatures of *T*^+^ and *T*^−^ populations underpinning AP polarization. We also show that this process is robust to modifications of aggregate size. Finally, transcriptomic comparison with the mouse embryo indicates that gastruloids develop similar mesendodermal cell types, despite initial differences in their primed pluripotent populations, which adopt a more mesenchymal state in lieu of an epiblast-like transcriptome. Hence, our findings suggest the possibility of alternate ESC states *in vivo* and *in vitro* that can converge onto similar cell fates.

## Teaser

**Highlighted Article:** Autonomous anteroposterior polarization in aggregates of mouse embryonic stem cells illustrates how alternative initial cell states between the embryo and the aggregates may converge onto similar fates.

## INTRODUCTION

The bilaterian body plan is established during early embryogenesis as part of a process commonly referred to as gastrulation, whereby collective cell rearrangements give rise to a multilayered blueprint with three germ layers (ecto-, meso- and endoderm) organized along three body axes: anteroposterior (AP), dorso-ventral (DV) and mediolateral (ML) (Keller et al., 2003; Leptin, 2005; Solnica-Krezel and Sepich, 2012; Sheng et al., 2021).

The mouse embryo is a prominent model system for mammalian development in which the AP axis is the first to be newly determined, whereas it has been suggested that DV axial polarity is technically inherited from the embryonic-abembryonic axis of the blastocyst (Takaoka and Hamada, 2012; Chen et al., 2018). Although the patterning of the mammalian early body plan is tightly coupled to biochemical and mechanical cues from extra-embryonic tissues (Arnold and Robertson, 2009; Rossant and Tam, 2009; Nowotschin and Hadjantonakis, 2010; Morris et al., 2012; Rivera-Pérez and Hadjantonakis, 2015; Stower and Srinivas, 2018), findings in (embryo-like) *in vitro* systems illustrate that AP axis formation can occur in an autonomous manner (Bedzhov et al., 2015; Shahbazi et al., 2019; Sagy et al., 2019; Baillie-Benson et al., 2020). In this context, gastruloids from aggregated mouse embryonic stem cells (mESCs) represent a prime example: they are grown without any localized signalling inputs, yet develop axial organization, congruent with the early mouse embryo (van den Brink et al., 2014; Turner et al., 2017; Veenvliet et al., 2020; van den Brink et al., 2020), displaying spatially delimited expression of marker genes for the AP, DV and ML axes, together with the emergence of distinct cell populations analogous to the three germ layers (Beccari et al., 2018).

This apparent ‘self-organizing’ capability of ESCs motivates the re-examination of *in vivo* development as a guided self-organizing process (Morales et al., 2021) under the influence of extra-embryonic tissues and species-specific mechano-chemical environment. Furthermore, *in vitro* systems, which are amenable to high-throughput imaging, transcriptomics and perturbation approaches, can provide insights into the principles of mammalian body plan formation that cannot be revealed by studying only the native embryo (Anlas and Trivedi, 2021; Moris et al., 2020; Steventon et al., 2021).

With respect to the AP axis establishment, the gastruloid system exhibits autonomous polarization of the transcription factor brachyury (or T) at the posterior end (Rivera-Pérez and Magnuson, 2005; Swalla, 2006; Petersen and Reddien, 2009), which has been interpreted as the first (system-wide) symmetry breaking event (van den Brink et al., 2014). Although it is known that this process relies on precisely timed interactions between Wnt/β-catenin and Nodal signalling (Turner et al., 2017), it remains unclear which molecular signatures at the cell level accompany the emergence of the AP axis.

Previous studies on the molecular characterization of gastruloids have mostly addressed the late stages after elongation when further differentiation and axial patterning has occurred after the application of Wnt agonist CHIR99021 (CHIR99) from 48-72 h post aggregation (hpa) (Beccari et al., 2018; van den Brink et al., 2020; Veenvliet et al., 2020). An emerging body of works on AP symmetry breaking has studied the formation of the T pole (Sagy et al., 2019; Rosen et al., 2022 preprint; Suppinger et al., 2023; McNamara et al., 2024; Mayran et al., 2023 preprint). A recent preprint mapped mesodermal and neural differentiation trajectories in gastruloids post CHIR99 application (i.e. after 72 hpa) (Rosen et al., 2022 preprint). Another comprehensive study reported that a binary response to this external cue exhibited by distinct, spatially segregated, pluripotent cell populations underlies AP symmetry breaking (Suppinger et al., 2023).

In accordance with the original works (van den Brink et al., 2014; Turner et al., 2017), the field relies on external Wnt stimulation to stabilize and enhance the polarization of T in gastruloids, which results in consistent elongation. This is particularly relevant because the initial mESC culture conditions vary across labs that grow cells in either two inhibitor (2i)-containing media (to promote a more uniform naive pluripotent state) or in serum and leukaemia inhibitory factor (LIF)-based media (ESL, containing primed subpopulations) (van den Brink et al., 2014; Turner et al., 2017; Veenvliet et al., 2020; van den Brink et al., 2020; Beccari et al., 2018; Rosen et al., 2022 preprint; Suppinger et al., 2023; McNamara et al., 2024; Mayran et al., 2023 preprint; Kolodziejczyk et al., 2015; Anlas et al., 2021). For the serum and LIF-based media, in particular, spontaneous polarization of T/Bra has been observed without CHIR99 in gastruloids, although with weaker expression (Turner et al., 2017), hinting at a self-organization process on its way that needs reinforcement from Wnt stimulation. However, the extent of concomitant tissue patterning of other cell types as cells exit pluripotency post-aggregation before and during CHIR99 application is, notably, still uncertain.

Here, we investigate the dynamics of AP axis establishment in 3D gastruloids, focusing on the differentiation and localization of the germ layers and cells denoting axial identity, concomitant with T polarization. Using Eomes and Aldh1a2 as spatially restricted markers for the anterior region, we assess the robustness of AP patterning in aggregates of varying sizes. Finally, we relate our findings to early lineage specification in the mouse embryo, identifying differences in the lineage priming and cell-adhesion state between mouse and gastruloid (primed) pluripotent stem cell populations.

## RESULTS

We employed a fluorescent reporter mESC line (T::GFP; Fehling et al., 2003) maintained in a KnockOut DMEM-based culture medium (ESL, see Materials and Methods for details) to monitor expression dynamics of *T*. As we were focusing on the first (AP) symmetry-breaking event, we followed the development of gastruloids until 72 hpa using the canonical protocol that employs a pulse of Wnt agonist CHIR99021 (CHIR99) from 48 to 72 hpa. (van den Brink et al., 2014; Anlas et al., 2021).

### Spatial restriction of germ layers in gastruloids is concomitant with T polarization

**Fig. 1.**
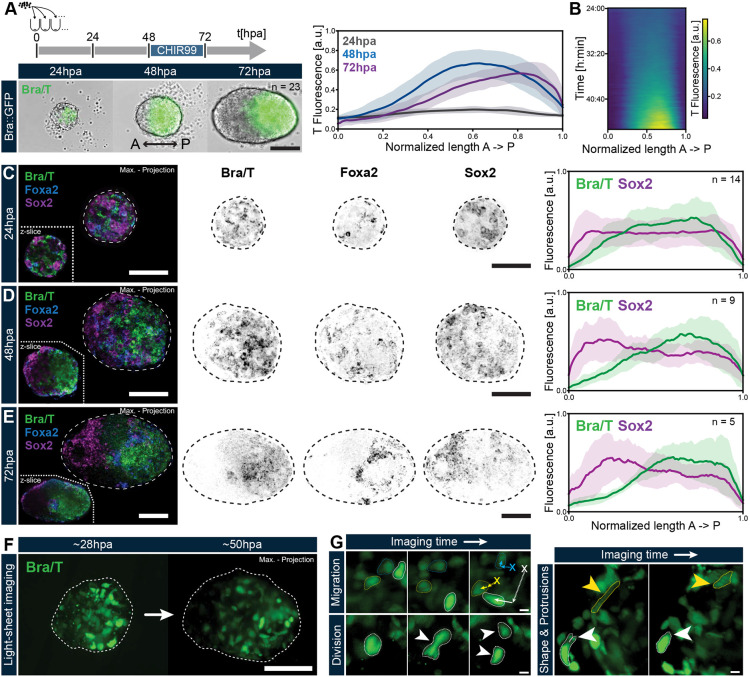
**Autonomous AP axis formation in gastruloids is demarcated by T polarization and accompanied by variable cell behaviours.** (A) Gastruloids were generated from T::GFP mESCs as previously described (Anlas et al., 2021), including a pulse of CHIR99 from 48 to 72 hpa. T symmetry breaking occurs by 48 hpa. Scale bar: 200 µm. Right panel displays T fluorescence intensity of the same *n*=23 gastruloids at 24, 48 and 72 hpa, plotted along the normalized AP axis length. Central lines indicate the mean intensity values of the included samples; surrounding shades mark the standard deviation. (B) Averaged kymograph of T expression along the AP axis of *n*=13 gastruloids, live imaged from 24 to 48 hpa, illustrates progression of T polarization over developmental time. (C-E) Hybridization chain reaction (HCR) *in situ* stainings for T, Foxa2 (anterior mesendoderm) and Sox2 (pluripotent and neural progenitors) in gastruloids at 24, 48 and 72 hpa, showing the arrangement of germ layer primordia pre- and post-T polarization. Scale bars: 100 µm. Right hand panels show quantifications of T and Sox2 fluorescence along the gastruloid AP axis (defined by localization of T expression) based on sum intensity projections of the HCR data for each timepoint. Data are mean±s.d. (F) Representative images of live imaging of gastruloids from T::mESCs, captured via LSFM showing the T polarization process and associated cellular dynamics. Aggregate shape was inferred via SIR-DNA counterstains. Scale bar: 100 µm. (G) Examples of cell behaviours displayed across LSFM datasets by T^+^  cells during aggregate-wide polarization. White arrowheads indicate cell protrusions; yellow arrowheads indicate cell shape change. Scale bars: 10 µm. The contrasts of the fluorescent channels have been adjusted for display purposes.

Upon aggregation of mESCs into gastruloids, transcriptional activity of *T* rapidly increases within a few hours, eventually leading to an evenly distributed expression throughout the aggregate between 24 and 48 hpa (Fig. S1A,B; Movie 1). Such a ubiquitous initial expression suggests that contact- or gravity-dependent *T* activation is not required at least for initiation of the symmetry breaking in gastruloids in contrast to previously reports for embryoid bodies (Sagy et al., 2019). Long-term imaging shows that, from a previously evenly distributed expression, the subtle asymmetry of *T* levels along the future AP axis emerges, which intensifies as reporter fluorescence shows further polarization in one half of the gastruloid (Fig. 1A,B). By 72 hpa, gastruloids consistently exhibit polarization of *T* expression that defines the posterior domain and is the site of incipient elongation (Fig. 1A). Importantly, in gastruloids from T::GFP mESCs grown in ESL, asymmetric *T* expression is already detectable by 48 hpa, before the pulse of CHIR99, similar to the observations made previously (Turner et al., 2017).

We then investigated germ layer formation and their relative spatial arrangement during early gastruloid development in 3D via *in situ* hybridization chain reaction (HCR) (Fig. 1C-E): At 24 hpa, in relation to *T* signal which may already show initial polarization, *Sox2* appears more homogeneous throughout gastruloids (Fig. 1C). As *T*^+^ cells become spatially restricted to the prospective posterior around 48 hpa, the highest expression of *Sox2* transcripts becomes mostly localized to the *T*^−^ region of the gastruloids (Fig. 1D). Quantification of expression along the AP axis (Fig. 1C-E, right panels) further shows that the spatial segregation of Sox2^+^ and *T*^+^ cells initiates before the application of CHIR99. Additional staining with phalloidin (F-actin) and GFP immunofluorescence (indicating T) do not indicate any obvious epithelial organization or any clear differences in cell shape, size or T expression in the core versus peripheral regions (Fig. S1D,E). By 72 hpa, the most elevated *Sox2* and *T* signals remain largely non-overlapping, thereby marking the anterior and posterior halves of the gastruloids, respectively (Fig. 1E; Fig. S1G). Furthermore, *T* and *Sox2* co-expressing regions at the posterior part of the gastruloids may potentially represent neuromesodermal progenitors (NMPs) (Tzouanacou et al., 2009).

*Foxa2* transcripts (anterior mesendoderm; Kimura-Yoshida et al., 2007; Burtscher and Lickert, 2009; Bardot et al., 2017) are sparsely dispersed throughout the aggregate until 48 hpa and do not seem to be predictive of T polarization, unlike previous findings in embryoid bodies (Sagy et al., 2019) (Fig. 1C; Fig. S1C). By 72 hpa, i.e. after symmetry breaking, *Foxa2* tends to be localized in the region anterior to the T domain, in accordance with its role as a marker of the anterior primitive streak (Fig. 1E).

Consistent with previous results (Turner et al., 2017), we observe that gastruloids grown from ESL can robustly undergo symmetry breaking in absence of the CHIR99 pulse (Fig. S1F). Furthermore, we find that, although *T* expression is weaker in such gastruloids, their growth, as well as initial elongation by 72 hpa, are similar to the controls. However, *Sox2* expression in these untreated aggregates has larger overlap with the *T*^+^ posterior region and thus shows less anterior restriction at 72 hpa when compared with the controls (Fig. S1F,G). Overall this argues that, although the CHIR99 pulse provides robustness to axial patterning, likely by promoting T^+^ mesodermal fates and diminishing pluripotent and/or neuroectodermal fates, the processes of germ layer differentiation and AP symmetry breaking are already in progress before the pulse.

### Diversity of motility and cell shape changes during symmetry breaking in gastruloids

To observe the symmetry-breaking event and associated cell behaviours at higher resolution, we imaged gastruloids using light sheet fluorescence microscopy (LSFM) (Huisken et al., 2004) (Movie 2). AP polarization is accompanied by aggregate-wide gain and loss of *T* expression, concomitant with highly motile behaviour of *T*^+^ cells, indicating that a combination of sorting and signalling may lead to polarization. Indeed, a recent study has demonstrated that the cell-specific coordination of a cadherin switch is needed for patterning in gastruloids (Mayran et al., 2023 preprint). A detailed look at the *T*^+^ cells shows a rich diversity in behaviours (Fig. 1F,G): extensive motility associated with frequent neighbour exchange, cell divisions and drastic shape changes from rounded to highly elongated cells, including dynamic formation and retraction of protrusions. Notably, migration speeds and overall displacements may vary among *T*^+^ cells (Fig. S1I).

Coarse-grained optical flow analysis (see Materials and Methods; Hashmi et al., 2022) of the *T* signal in our LSFM data broadly depicts two main domains of opposite tissue flows concomitant with the emergence of a T^+^  pole (Fig. S1H): flows of *T*^+^ cells towards the prospective posterior region and flows of cells with lower *T* expression away from the posterior tip. Overall, such large scale flows could enhance the polarization (Gsell et al., 2023 preprint) and likely build on differential tissue adhesion and convergent extension that have been recently shown to explain the shapes of elongating gastruloids (de Jong et al., 2024).

### Analysis of cell types and populations around the symmetry-breaking event

**Fig. 2.**
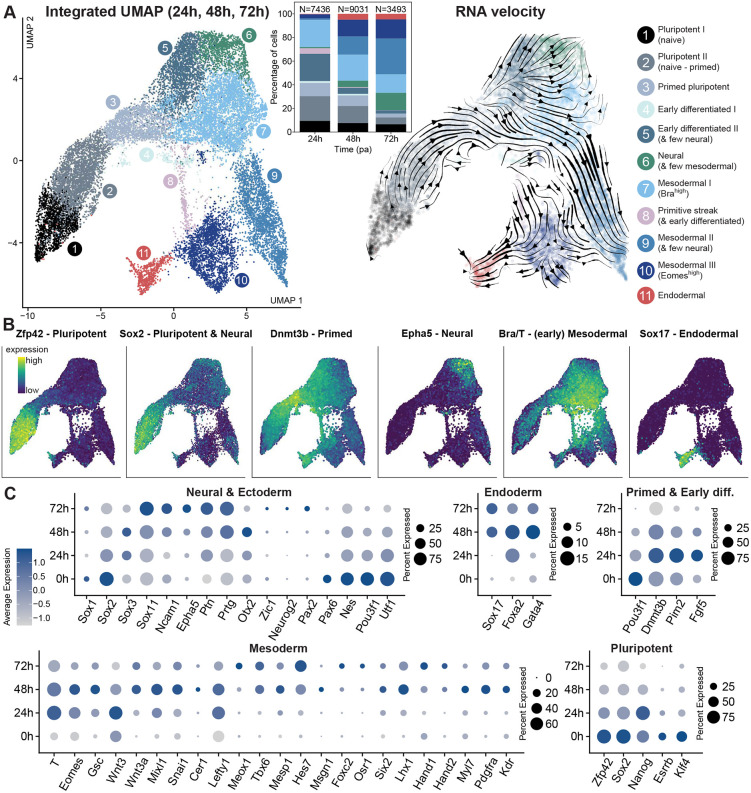
**Single-cell transcriptomic analysis of early gastruloid development highlights early, autonomous lineage segregation.** (A) Germ layer emergence in early gastruloids (24-72 hpa) delineated by scRNAseq. Left: UMAP plots with clusters annotated according to germ layer or cell state identity are displayed. Middle: stacked bar charts illustrate germ layer differentiation dynamics in gastruloids. Right: RNA velocity analysis on the integrated UMAP delineates differentiation trajectories in gastruloids. (B) Expression of key marker genes for pluripotent, neural, primed, mesodermal and endodermal cells in the integrated UMAP illustrates the spatial segregation of germ layers. (C) Expression of several genes denoting neural, endodermal, primed and early differentiation, mesodermal, and pluripotent tissues are depicted for each developmental time point. Sizes of circles indicate the fraction of expressing cells; color code indicates the averaged expression level for a given gene.

The AP axis generated by the T symmetry-breaking event has, so far, mostly been characterized in terms of a few marker genes for germ layer progenitors or in gastruloids from mESCs grown in 2i-based medium (Suppinger et al., 2023), whereby the dynamics of T emergence (both timing and spatial localization) differ from those studied here, made from cells cultured in ESL (as per the original protocol; van den Brink et al., 2014; Turner et al., 2017). Therefore, we performed single-cell RNA sequencing (scRNA-seq) of mESCs grown in ESL, representing the 0 hpa timepoint, and of gastruloids at 24, 48 and 72 hpa (Fig. 2A,B; Figs S2-S4; Tables S1-S3).

At 0 hpa, distinct pluripotent, primed pluripotent and early differentiated cell populations were detected, highlighting the presence of a spectrum of pluripotent states in mESCs grown in ESL without additional small molecule inhibitors (Kolodziejczyk et al., 2015) (Fig. S2A,B). The early differentiated populations are distinguished from primed pluripotent cells by decreased (but still detectable) expression of primed pluripotent state markers (*Dnmt3b* and *Pim2*), concurrent with (comparatively) increased levels of early germ layer differentiation factors (e.g. *T*, *Fgf5* and *Eomes*).

For comparison, we performed integration of our T::GFP mESCs at 0 hpa with a previously published dataset from E14 wild-type mESCs (Alda-Catalinas et al., 2020) (Fig. S2C). The T::GFP mESCs overlap well with the wild-type E14 mESCs; however, they exhibit a substantially higher percentage of cells forming primed and early differentiated clusters. These distinctions in ratios of such subpopulations likely arise from individual cell line variability (Cahan and Daley, 2013), but also media composition, despite the same designation [i.e ĖSL made from GMEM (van den Brink et al., 2014; Turner et al., 2017; Beccari et al., 2018) versus DMEM (Alda-Catalinas et al., 2020; Rosen et al., 2022 preprint) or KO-DMEM (Sagy et al., 2019; this work)]. Altogether, the abundance of primed pluripotent cells in the 0 hpa dataset suggests that a significant subset of T::GFP mESCs grown in ESL are already on the threshold of pluripotency exit and germ layer specification. This is consistent with the rapid formation of mesoderm in gastruloids, seen as a swift emergence of aggregate-wide T activity during live imaging (Fig. S1A).

On the other hand, our aggregate datasets from 24, 48 and 72 hpa, when integrated, cluster distinctly into populations indicating mESC differentiation from naive pluripotent to primed and early differentiated states, (anterior) primitive streak-like populations and those corresponding to the three germ layer identities (Fig. 2A,B; Fig. S3A-E; Fig. S4; Table S2). As expected, the proportion of naive and primed pluripotent, as well as early differentiated cells diminishes from 24 hpa to 72 hpa (Fig. 2A, inset; Fig. S2D), consistent with previous observations (Turner et al., 2017). Although the endodermal fraction remains almost identical between 48 and 72 hpa, more differentiated mesoderm subtypes (mesodermal II and III) as well as neural fractions increase in this timespan, concomitant with a decrease of early *T*^+^mesoderm (mesodermal I).

RNA velocity analysis on the integrated dataset (Fig. 2A, right panel) further corroborates a lineage progression from pluripotent to primed populations to more differentiated groups: primed pluripotent (high *Dnmt3b*, *Pim2* and *Mkrn1*) and early differentiated I regions (high *Cbx2*, *Lin28a* and *Scd2*) lead – in case of the former via early (*T*^+^) mesodermal cells (mesodermal I, high *T*, *Fgf8* and *Cdx2*) – towards the mesodermal II cluster (high *Rspo3, Cited1* and *Aldh1a2*). In parallel, separate trajectories from the early differentiated II population (high *Pim2*, *Fst*, *Epcam* and *Bex1*) head towards neural-like cells [high pleiotropin (*Ptn*), *Nkx1-2*, *Ncam1* and *Epha5*) and the mesodermal I cluster whereby some streamlines further advance to the mesodermal II population. The cluster corresponding to (likely anterior) primitive streak-like cells (high *Goosecoid, Mixl1, Apln*) mostly progresses via anterior mesodermal (mesodermal III, high *Eomes, Lhx1, Mesp1*) towards *Sox17*^+^  endodermal populations.

### Dynamics of marker expressions and proportions of cell populations during symmetry-breaking

Our scRNASeq data of the earliest stages of gastruloid development reveal that key developmental events, such as the onset of T expression and definitive endoderm emergence, are already underway prior to CHIR99 application (i.e. in absence of external Wnt stimulation). In contrast to the former assessment of germ layer specification via bulk RNA sequencing (Beccari et al., 2018), we were able to probe the temporal progression of the proportion and expression profiles of specific cell types (Fig. 2C) and related these findings to the clustering results.

Genes such as *T, Eomes, Mixl1* and *Lefty1* begin to display elevated and pervasive expression by 24 hpa, around the onset of germ layer formation in gastruloids (Fig. 2C, “Mesoderm”), roughly linking this timepoint in our culture condition to the E6.5 mouse embryo (Tam and Loebel, 2007). It is possible that this timeline can be shifted across different culture conditions depending on the abundance of primed pluripotent cells in the starting population as mentioned before. Early posterior mesodermal cells (*T*^*high*^ *Eomes*^*low*^, mesodermal I cluster) as well as few anterior mesodermal or mesendodermal populations (*Eomes*^*high*^ *T*^*low*^, mesodermal III cluster) are also already present at 24 hpa (Fig. 2A,C). In addition, *Foxa2* surges at 24 hpa, consistent with its role as an early determinant of anterior mesendodermal fate at the onset of gastrulation.

Already by 48 hpa, the anterior primitive streak population diminishes and endodermal markers (*Sox17* and *Gata4*) surface. This suggests that, akin to the mesoderm, the definitive endodermal lineage can also arise in an autonomous manner (i.e. without external signalling pathway modulation) in our culture condition (Fig. 2A-C). Furthermore, mesodermal cells start differentiating into subpopulations (mesodermal II and III clusters), distinguished by increasing expression of marker genes for paraxial (or presomitic), intermediate and lateral plate mesoderm, such as *Tbx6*, *Hes7*, *Msgn1*, *Osr1*, *Lhx1*, *Meox1*, *Hand1* and *Mesp1*. At the 72 hpa timepoint, cardiac mesoderm-like populations (*Hand1*^+^  and *Hand2*^+^) emerge, together with a distinct, albeit very small, subset of vascular and endothelial progenitors (*Egfl7*^+^), both of which could be resolved as individual clusters upon analysis of the isolated 72 hpa dataset (Fig. S2F,G,I; Table S3). Notably, a recent study demonstrated how gastruloids can be coaxed into recapitulating early heart morphogenesis by 168 hpa via application of cardiogenic factors that are likely enriching these progenitors (Rossi et al., 2020).

Likewise, neural(-like) cells also begin to appear at 48 hpa, as shown by upregulation of neuronal differentiation factors, including protogenin (*Prtg*), *Sox11*, *Epha5* and *Ncam1*, although definitive marker *Sox1* displays very low expression until 72 hpa, remaining limited to a small subset of cells (<10%) (Fig. 2C; Fig. S2E). It is worth mentioning that the mesodermal II cluster also features a very small subpopulation of *Sox1*^+^  cells, arguing for neural admixture. In general, some early neural lineage markers are highly and pervasively expressed at 0 h, which could be explained by the default neural priming of early differentiating cells in standard 2D culture. Expression of some of these neuroectodermal genes thereupon mostly diminishes (e.g. *Nes*, *Utf1*, *Sox2*, *Pou3f1* and *Pax6*), whereas others reach their peak by 72 hpa (*Ptn*, *Prtg*, *Pax2*, *Epha5*, *Ncam1*, *Zic1*, *Neurog2*, *Sox1* and *Sox11*). We could not identify obvious anterior neural populations, in line with previous findings in ‘default’ gastruloids.

### Spatial restriction and axial pattering of germ layers initiate before external Wnt stimulation

Consistent with the average expression analyses (Fig. 2C), UMAPs show the distribution of endodermal and neural lineages at the single cell level and corroborate that, by 48 hpa, distinctly clustered germ layer populations have already emerged (Fig. S5A). Such a segregation of cells expressing *Zfp42* (pluripotent), *Epha5* (neural), *Tbx6* (paraxial mesoderm), *Mesp1* (cardiac, haematopoietic and myogenic mesoderm) and *Sox17* (definitive endoderm) reflects AP axial organization that was previously only reported in elongated gastruloids at 96-120 hpa (Turner et al., 2017; Beccari et al., 2018).

**Fig. 3.**
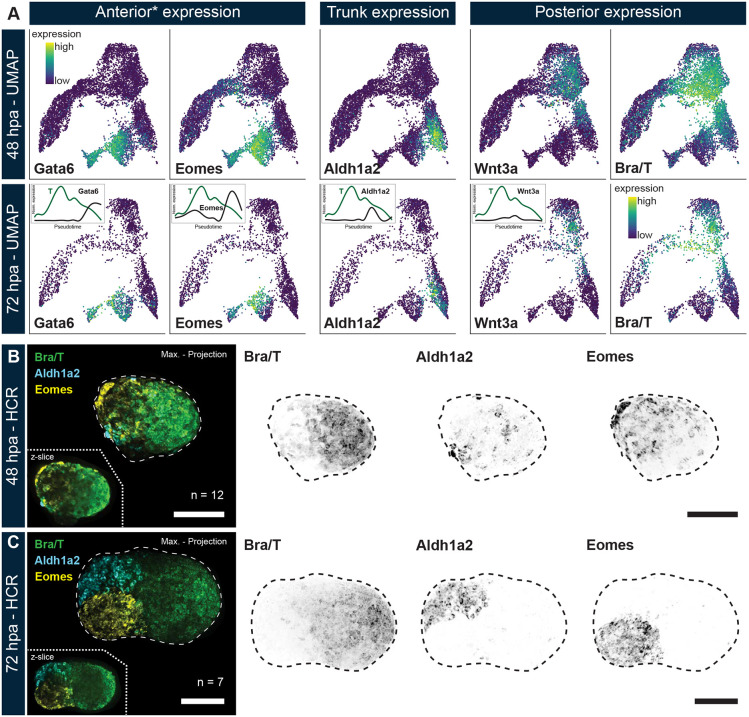
**Spatial AP axial patterning in gastruloids before external Wnt stimulation.** (A) Expression of marker genes for anterior (asterisk indicates anterior only in gastruloids, due to absence of cranial patterning), trunk and posterior tissues on the integrated (24, 48 and 72 hpa) UMAP that is filtered for cells from only 48 and 72 hpa. Segregation of expression clusters occurs already at 48 hpa, before external CHIR99 application. Insets in the 72 hpa UMAPs display normalized T (as a reference) and AP marker gene expression plotted along inferred (developmental) pseudotime based on the integrated scRNA-dataset. (B,C) Hybridization chain reaction (HCR) *in situ* for key AP axial and germ layer marker genes T (posterior, early mesoderm), Aldh1a2 (trunk tissues) and Eomes (anterior mesendoderm) at 48 (B) and 72 (C) hpa. Spatial segregation of expression patterns is initiated by 48 hpa. Scale bars: 100 µm. The contrasts of the fluorescent channels have been adjusted for display purposes.

We then assessed expression of additional key AP axial patterning genes: *Gata6* and *Eomes* (anterior mesendoderm), *Aldh1a2* (tissues lateral to the primitive streak and the node), and *Wnt3a* and *T* (posterior, tailbud mesoderm) to probe the extent of axial patterning before the pulse of Wnt agonist CHIR99 (Fig. 3A; Fig. S5B). UMAPs show that, by 48 hpa, these markers are already restricted to distinct cell populations. *Gata6* and *Eomes* transcripts are abundant in the endodermal and anterior mesodermal (mesodermal III) clusters, which is in contrast to previous reports based on bulk RT-PCR, where the first expression of *Gata6* was only detected around 96 hpa (Turner et al., 2017). On the other hand, the patterning factor *Aldh1a2* is upregulated in the newly emerged differentiated mesoderm (mesodermal II cluster). Last, *Wnt3a* mostly exhibits expression within the *T*^+^  early mesodermal group (mesodermal I cluster).

We then performed pseudotime analysis (Qiu et al., 2017; Cao et al., 2019) on our integrated dataset (Fig. 3A, insets) to probe expression dynamics of these genes relative to *T*. Distinct (pseudo)temporal segregation can be observed with *Gata6* and *Eomes* representing ‘late-peaking’ genes – although *Eomes* also has a notable initial peak that is consistent with its role in specifying the incipient mesoderm (Tosic et al., 2019; Schröder et al., 2023 preprint) – while *Aldh1a2* and *Wnt3a* exhibit successively ‘earlier’ expression maxima. This does not necessarily imply that (in particular) Aldh1a2 is always expressed before Gata6 in actual developmental time; however, it substantiates the presence of distinct differentiation paths and resulting cell type identities associated with either (gastruloid) anterior or trunk tissue.

Notably, these UMAP-based inferences further translate into spatial restriction of *T*, *Aldh1a2* and *Eomes*, as seen in HCR stainings of gastruloids at 48 hpa (Fig. 3B). Such a level of organized differentiation of mESCs into restricted spatial territories has not been demonstrated before at such an early developmental stage in the absence of external patterning cues. This segregation of cell populations is further refined at 72 hpa after CHIR99 application (Fig. 3C), but can also occur without Wnt agonist treatment (Fig. S5C). Moreover, separation of *T* versus *Aldh1a2* and *Eomes* is more evident, i.e. there is less overlap, when compared with the separation of *T* versus *Sox2* expression domains (Fig. 1E; Fig. S1G). Consequently, these results corroborate that spatial restriction of germ layers can not only initiate before Wnt stimulation but also proceed without the need for such an external cue.

### T polarization and axial organization are robust to changes in aggregate size

**Fig. 4.**
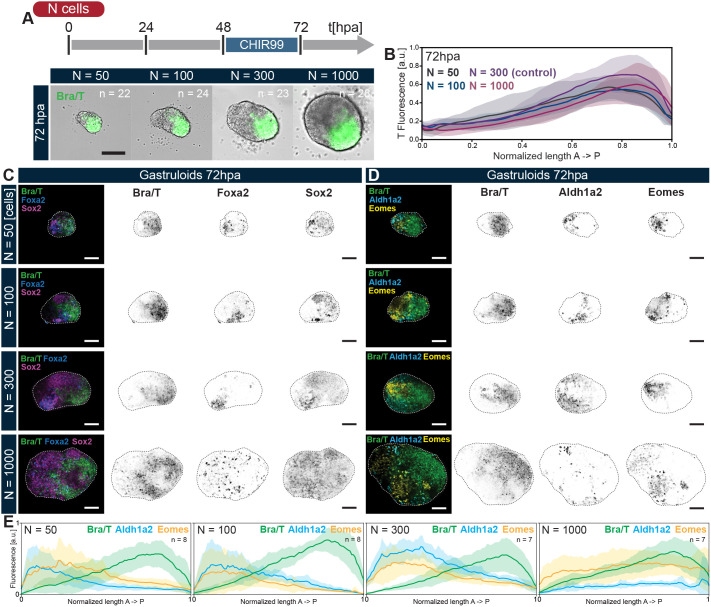
**T polarization and AP axial patterning are robust to changes in aggregate size.** (A,B) Representative images (A) and quantitative analyses (B) of T expression along the normalized AP axis of gastruloids grown from different initial cell numbers (N) demonstrates the reproducibility of T symmetry breaking and scaling of relative T domain size between 50 and 1000 starting cells. (C,D) Hybridization chain reaction (HCR) *in situ* of gastruloids from different initial cell numbers at 72 hpa for key germ layer markers and AP axial patterning genes T, Foxa2 and Sox2 (C), as well as T, Aldh1a2 and Eomes (D). Scale bars: 100 µm. The contrasts of the fluorescent channels have been adjusted for display purposes. (E) Quantification of AP patterning gene expression along the gastruloid AP axis (defined by localization of T) based on sum intensity projections of HCR data from D. The segregation of expression domains is robust, except for *N*_*i*_ = 1000. Data are mean±s.d.

In order to probe the robustness of this displayed transcriptional AP patterning to aggregate size, we generated gastruloids from *N*_*i*_ = 30, 50, 100, 300, 1000 or 2000 cells. At *N*_*i*_ = 50-1000, T polarization occurs reproducibly and gastruloids show similar relative area growth until 72 hpa (Fig. 4A,B; Fig. S6A-C). Gastruloids across these sizes display similar proportion of T^+^  domain relative to the length of their AP axes. However, large gastruloids (> *N*_*i*_=1000) can have multiple *T* poles, whereas very small ones (*N*_*i*_=30) are heterogeneous, some of which show little to no growth, elongation or T expression (Fig. S6B,C). In this context, although we only focus on gastruloids up the 72 hpa timepoint, a recent study showed precise scaling of several gene expression domains in late (120 hpa) gastruloids of varying sizes (Merle et al., 2024).

We further looked into the extent of axial organization, beyond polarization of T, in aggregates of different sizes through HCR staining (Fig. 4C,D; Fig. S6D,E). Similar to the control case (*N*_*i*_=300), gastruloids made from 50, 100 or 1000 cells undergo mesendoderm differentiation, as demonstrated by spatially delimited expression domains of *T*, *Foxa2* and *Sox2* (Fig. 4C; Fig. S6D). Remarkably, *Aldh1a2* and *Eomes* also display clear and reproducible AP axial segregation from the *T*^+^  domain by 72 hpa (Fig. 4D; Fig. S6E,F), as highlighted by averaged expression analysis of the HCR data (Fig. 4E; Fig. S6F, right panel). Conversely, in large aggregates, multiple *T*^+^ poles may be observed, also resulting in less conspicuous separation between *T*, *Eomes* and *Aldh1a2* gene expression domains, arguing that the upper limit for proper AP axis specification lies at around *N*_*i*_=1000 (Fig. 4E).

To summarize, our data suggest that 3D gastruloids can generate different cell types and, in particular, all germ layer progenitors within a certain size range, in contrast to the observations made in their planar/2D counterparts confined on micropatterns (Warmflash et al., 2014; Morgani et al., 2018). The scaling of the T pole in gastruloids across sizes argues for a self-organizing mechanism that cannot feature a fixed length scale, as in a classical Turing pattern (Umulis and Othmer, 2013). It must be noted that this potential for generating all sub-populations at the earliest stages does not rule out the possibility of size-dependent enrichment of lineage-specific cells types, as observed in embryoid bodies (Hwang et al., 2009).

### Different cell states between T^+^  and T^−^  underpin the polarization event

Given the robustness of T polarization and the emergence of distinct cell states in gastruloids, we aimed at probing differences between *T*^+^ and *T*^−^ cell populations that could underpin the tissue flows and symmetry breaking at the tissue level, similar to observations in other systems (Krieg et al., 2008; Jeffrey et al., 2021; Hashmi et al., 2022; Gsell et al., 2023 preprint). Global analysis of the expression dynamics of cell-adhesion and cytoskeletal genes shows that changes in these occur concurrently with T symmetry breaking (Fig. S7A). *Cdh1* (E-cadherin) downregulation is concomitant with upregulation of *Cdh2* (N-cadherin) as the T pole is formed by 48 hpa, thus revealing cadherin switching, a hallmark of germ layer establishment (Acloque et al., 2009; Lamouille et al., 2014). Conversely, epithelial markers keratin 8 (Krt8) and Krt18 are highly expressed in mESCs at 0 h but significantly downregulated in gastruloids from 24 to 72 h. On the other hand, mesenchymal genes vimentin (*Vim*) and fibronectin (*Fn1*) exhibit increased expression both in mESCs as well as in aggregates at 48 hpa and 72 hpa. Their abundance in the 0 h dataset may be explained by Vim also playing a role in early (neuronal) differentiation of cultured mESCs (Pattabiraman et al., 2020) and Fn1, in turn, being crucial for their self-renewal (Hunt et al., 2012). Altogether, aforementioned observations are consistent with and add to previous findings (Beddington et al., 1992; Turner et al., 2014; Mayran et al., 2023 preprint).

**Fig. 5.**
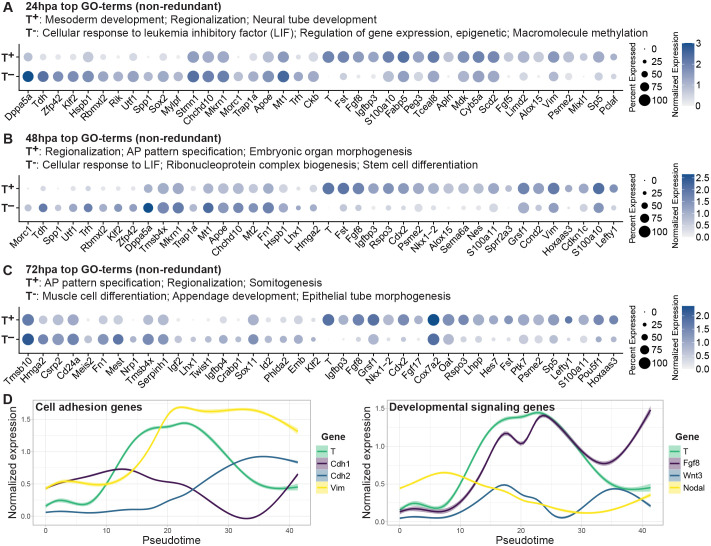
**T**^+^  **and T**^−^  **populations exhibit distinct transcriptional cell states that progress along developmental time.** (A-C) Single cell transcriptomic comparison of T^+^  with T^−^  cell populations around the symmetry-breaking event at 48 hpa. The top three non-redundant GO terms, as well as the top 20 most differentially expressed genes are shown for each population across three timepoints (24, 48 and 72 hpa). For each gene, the size of the circle indicates the fraction of expressing cells; the color code indicates the (normalized) expression level in those cells. T^+^ populations upregulate genes associated with cell motility and adhesion. (D) Normalized expression of key cell adhesion (left) and developmental signalling (right) genes with T plotted along pseudotime, inferred from the integrated scRNA-dataset featuring only the 24 and the 48 hpa timepoints. Data represent the regression with 95% confidence interval (shaded region, as describe in the Materials and Methods).

Computational segregation and analysis of *T*^+^ and *T*^−^ populations from our gastruloid scRNA-seq dataset shows that *T*^+^ cells at all developmental timepoints (24 hpa, 48 hpa and 72 hpa) are indeed the primary source of elevated expression of genes implicated in cell migration and adhesion, including *Vim*, *S100a10*, *S100a11*, *Igfbp3*, *Ptk7*, *Apln*, *Lefty1*, *Pitx2*, *Sema6a*, *Mixl1* and *Fgf8* (Fig. 5A-C) (Nakaya and Sheng, 2008). Additionally, pseudotime analyses of our 24 and 48 hpa (i.e. before the CHIR99 pulse) datasets clearly show a cadherin switch associated with peaking *T* expression across cells in the gastruloids (Fig. 5D, left panel). Likewise, increasing *Vim* expression closely follows the rising *T* levels (pseudo-temporally) and is maintained as aggregate-wide cell differentiation progresses.

Regarding developmental genes, *T*^+^cells (as expected) upregulate genes involved in early AP patterning and mesoderm specification processes, such as *Wnt*, *Map2k1*, *Mapk1* and *Tgfb* signalling effectors, as well as their modulators (e.g. *Cdx2*, *Sp5*, *Fgf8*, *Fgf17*, *Nkx1-2*, *Igfbp3*, *Fst*, *Rspo3* and *Lefty1*; Fig. 5A-C; also see Tables S4-S9). Furthermore, relative to *T*, key morphogens *Fgf8*, *Wnt3* and *Nodal* exhibit peak expression at different points along the inferred differentiation pseudotime axis (Fig. 5D, right panel), again arguing for distinct *T*^+^ and *T*^−^ cell (sub-)state transitions before CHIR99 application.

Conversely, the majority of *T*^−^ cells upregulate pluripotency as well as early neuroectodermal lineage markers, such as *Zfp42*, *Dppa5a*, *Sox2* and *Utf1*. At 48 hpa, the *T*^−^ population further exhibits elevated expression of anterior mesendodermal genes (*Lhx1* and *Trh*) (Fig. 5B) and, by 72 hpa, also of anterior mesodermal and neural determinants (*Nrp1*, *Meis2*, *Id2* and *Crabp1*) (Fig. 5C). Gene ontology (GO) enrichment terms for *T*^+^ and *T*^−^ cells further substantiate these findings (Tables S4-S9).

Similar analyses of *Sox*2^+^ and *Sox*2^−^ populations at different timepoints demonstrate that many upregulated genes in *Sox*2^−^ cells correspond to those in *T*^+^ populations, and vice versa, particularly at 24 hpa. This indicates that the two main tissue fates in gastruloids during the first developmental day arise from mesodermal and pluripotent and/or neuroectodermal lineages (Fig. S7B-D; Tables S10-S15).

### Integration of scRNA-Seq data from gastruloids and early mouse embryos allows comparison of differentiation trajectories

**Fig. 6.**
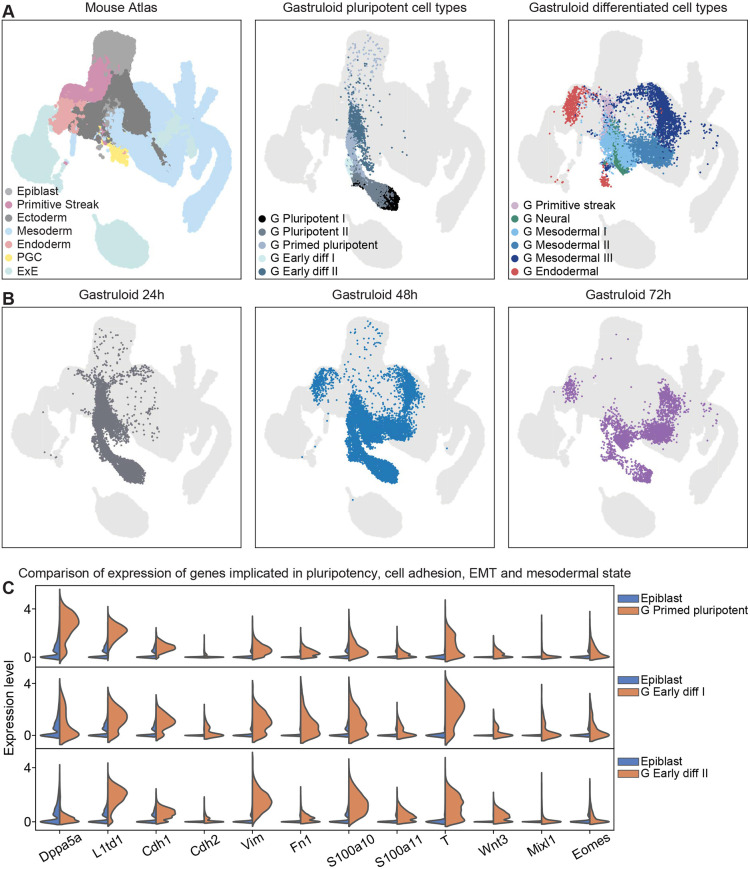
**The comparison of cell states and germ layer identities between gastruloids and the early mouse embryo highlights differences in developmentally early populations.** (A) UMAP plots displaying MNN-based integration of a comprehensive mouse sc-atlas (Pijuan-Sala et al., 2019) from E6.5 to E8.5 with the gastruloid datasets of 24, 48 and 72 hpa. Cell types were labelled according to germ layer identity or differentiation state to identify convergent populations. There is substantial overlap of mesodermal II and III, and endodermal gastruloid clusters to cells from the native embryo, but little intersection of less differentiated and neural or neuroectodermal populations with corresponding mouse cell types. (B) UMAPs are plotted as above; however, gastruloid cells are filtered by developmental day instead of germ layer identity. (C) Expression of marker genes for pluripotency, cell adhesion, EMT and mesodermal state, as well as general early differentiation compared between mouse epiblast and corresponding gastruloid populations (primed pluripotent, and early differentiated I and II).

We then sought to address to what degree the differentiation dynamics *in vitro* displayed by ESCs developing in the absence of localized external (or extra-embryonic) signals are similar to the process *in vivo*. Although most previous research efforts have focused on mapping the developmentally late cell types in gastruloids to their respective *in vivo* counterparts (Beccari et al., 2018; van den Brink et al., 2020; Veenvliet et al., 2020; Berenger-Currias et al., 2022), early germ layer formation has not been this deeply scrutinized. Such a comparison is also important, given that distinctions in spatial patterning and timing of AP polarization appear to depend on the initial ESC population used (ESL in our case versus 2i+LIF in another recent study; Suppinger et al., 2023). Hence, we integrated our gastruloid scRNA-seq dataset from 24 to 72 hpa with a previously published mouse sc-atlas (E6.5-8.5) (Pijuan-Sala et al., 2019) (Fig. 6A,B; Fig. S8A-D). To enable a direct comparison, we assigned broad germ layer and cell state identities to mouse sc-atlas cell types, thereby coarse-graining the available detailed annotation.

A key caveat of the conventional integration algorithm is the assumption that the cell types between the datasets of interest are basically equivalent and thus reliable label transfer can be performed. For assessing the validity of such an approach, we therefore computed various quality control (QC) parameters (Fig. S8E-G, see Materials and Methods) and found several inconsistencies, arguing that at least some populations in the mouse embryo and the early gastruloid may, in fact, not be similar enough. Hence we opted to perform a more unbiased comparison between the scRNA-seq datasets through mutual nearest neighbour (MNN)-based integration (Haghverdi et al., 2018).

The resulting UMAPs illustrate that differentiated mesodermal and endodermal populations from gastruloids map well onto corresponding mouse clusters (Fig. 6A). However, gastruloid neural-like cells do not align with the mouse neuroectodermal population, possibly owing to the early gastruloids lacking true neuroepithelia, despite the expression of neural markers such as *Sox1*, *Epha5* and *Ncam1*. Separating integrated gastruloid datasets by developmental time points distinctly shows the lineage progression from primed pluripotent, primitive streak and early mesodermal cell types to definitive endodermal and more-differentiated mesodermal population (Fig. 6B), consistent with previous results (Fig. 2).

### Germ layer differentiation in gastruloids occurs through developmental trajectories distinct from those in the mouse embryo

Strikingly, gastruloid clusters comprising the less lineage-committed populations (pluripotent, primed pluripotent and early differentiated) also show little overlap in the UMAP with the mouse epiblast (Fig. 6A). This remained the case even when the reference atlas was filtered to include only epiblast cells (Fig. S9A) and when a separate mouse dataset (Nowotschin et al., 2019), also spanning pre-gastrulation development (E3.5-5.5), was employed for integration (Fig. S9B). Further analysis of most variable genes between mouse (Pijuan-Sala et al., 2019) and gastruloid populations shows clear differences between them at the transcriptional level (Fig. S9C). Gastruloid primed pluripotent cells upregulate pluripotency maintenance factors such as *Dppa5a, L1td1* and *Rif1* as well as lipid metabolism-related genes *Scd2*, *Hmgcs1* and *Acsl3*. On the other hand, mouse epiblast markers mostly encompass components of other metabolic processes, namely glycolysis and fermentation (*Gapdh*, *Pgk1* and *Ldha*), ATP generation (*Cycs*), and iron and pH homeostasis (*Ftl1* and *Car4*, respectively), and the chromatin remodeller *Hmgb1* and the actin cytoskeleton modifier *Pfn1*. Conversely, gastruloid early differentiated I and II clusters exhibit increased expression of cell migration and overall mesenchymal state markers (*Prtg*, *Enah*, *Fgfr1*, *Vim* and *S100a10*). In turn, the epiblast population, in addition to the above-mentioned transcripts, shows heightened expression of other metabolic and energy homeostasis-related factors, such as *Pkm*, *Aldoa*, *Selenow* and *Ubb*.

To further delineate distinctions in transcriptional state and lineage specification dynamics of mouse epiblast versus gastruloid primed pluripotent and early differentiated clusters, we analysed expression of selected genes for (early mesendodermal) differentiation (*T*, *Wnt3*, *Mixl1* and *Eomes*), and for cell adhesion, motility and mesenchymal state (*Cdh1*, *Cdh2, Vim*, *Fn1*, *S100a10* and *S100a11*) (Fig. 6C). In general, these transcripts exhibit higher and more-pervasive expression in gastruloid cells when compared with epiblast cells, as seen by the broader histograms. This trend is even more pronounced in early differentiated populations, for the cell-adhesion and motility genes as well as for T, which is indicative of the onset of cadherin switching in these cells.

In summary, this argues that, upon exiting naive pluripotency, gastruloid-forming mESCs from ESL immediately adopt a mesenchymal-like identity, with substantial co-expression of the early (mesodermal) lineage priming factor *T*, thereby comprising a cell-adhesion (and metabolic) state that is distinct from the epithelial mouse epiblast. This may also help to explain the comparatively poor mapping of gastruloid neural-like cells to the *in vivo* equivalent, as these appear to be generated mostly by non- or ‘semi-epithelial’ progenitors. Although eventually the differentiated mesodermal and endodermal populations from gastruloids map well onto corresponding mouse cell types, they also have a transcriptional similarity despite originating from cells harbouring a different (transcriptional) state than the native epiblast.

## DISCUSSION

AP axis formation in embryos results from tight coordination between extra-embryonic and embryonic tissues. Conversely, *in vitro* systems, such as gastruloids, provide insights into the inherent capacity of ESCs to generate (at least) a primary body axis.

We report the earliest stages of AP polarization in gastruloids, demarcated by spatial segregation of *T* (posterior mesoderm) versus *Aldh1a2* and *Eomes* (trunk and anterior mesoderm) alongside *Sox2*^*high*^ (neuroectoderm and pluripotent) domains. This process is robust against changes in cell number and initiates before external Wnt activation (in cells from ESL media). Furthermore, the extent of axial patterning within the first 72 hpa highlights the remarkable developmental potential of gastruloids beyond a simple collection of cells representing a posterior growth zone of the mouse embryo (Rossant and Tam, 2021; Rossant and Tam, 2022).

A recent study that mapped differentiation via single gastruloid, single cell RNA-sequencing showed a mesodermal or neural bias in gastruloids based on Wnt signalling response (Rosen et al., 2022 preprint). Understanding such later differentiation signatures demands an in-depth characterization of their earliest stages, which is the focus of our work here. As more research groups adapt gastruloids as a model system, it is important to appreciate how differences in developmental dynamics, particularly during the first 72 hpa, may arise due to 2D cell culture conditions of mESCs (2i or ESL medium and the basal media used for them) employed to generate aggregates. This is relevant when comparing data (e.g. transcriptomic) from different studies employing distinct cell lines and culture conditions.

For example, mESCs from 2i-based media establish the AP axis (opposing T and Sox2 domains) only between 72 and 96 hpa, following the CHIR99 pulse (Suppinger et al., 2023), starting from a core of Sox2^+^  core surrounded by a T^+^  peripheral population, which is in contrast to our observations derived from mESCs grown in ESL media. This is likely because cells in ESL are more heterogeneous, with a subset already on the threshold of exiting pluripotency (Kolodziejczyk et al., 2015). This can accelerate mesodermal differentiation and the overall timing of AP axis specification in gastruloids.

Studies on embryo-like *in vitro* systems commonly aim to present a faithful replica of native development and hence focus on highlighting similarities to the actual embryo. Here, we report key differences to the events observed *in vivo* that have not been thoroughly investigated previously. Although embryo-like meso- and endodermal cell types surface during germ layer emergence, neuroectodermal, pluripotent, primed pluripotent and early differentiating populations map comparatively poorly to the mouse epiblast.

The observation that early-primed epiblast-like cells (EpiLCs) and particularly epiblast SCs (EpiSCs) fail to generate proper, i.e. elongated, gastruloids (Cermola et al., 2021) is particularly intriguing in this context. In the case of a recent study that succeeded in generating ‘Epi-gastruloids’ (Girgin et al., 2021), this was achieved by aggregating naive pluripotent mESCs followed by pre-differentiation in 3D culture, which would arguably enable cells to better retain cell-to-cell adhesion and mesenchymal state, two key factors that are necessary for gastruloid formation. Such results may further corroborate that, at least when using the base gastruloid protocol, AP axis and concomitant germ layer specification cannot efficiently proceed through a direct 2D *in vitro* equivalent of the *in vivo* epiblast cell state.

The emergence of *in vivo*-like meso- and endodermal cell types in gastruloids from primed pluripotent populations that exhibit a more mesenchymal (adhesion-) state in lieu of an epithelial epiblast-like transcriptome suggests a convergence of cell fates between *in vivo* and *in vitro*, via potentially distinct morphogenetic and transcriptomic developmental trajectories. Notably, a recent study found multiple transcriptional trajectories during somitogenesis, indicating a certain molecular flexibility in early cell-type specification (Guibentif et al., 2021). Evidence of alternative developmental modes have been demonstrated in other metazoans (Anlas and Trivedi, 2021): For example, dissociated and re-aggregated *Nematostella* gastrulae develop germ layers through delamination, multipolar ingression and cavitation, instead of invagination (Kirillova et al., 2018).

Such alternative developmental modes have not been explicitly characterized in a mammalian model. Future studies with mESC-based structures may further elucidate these and the mechanisms by which the different differentiation trajectories proceed. *In vitro* systems facilitate integration of engineering approaches, data science and theoretical modelling to make testable predictions (Gritti et al., 2021). As a result, they can provide insights into the developmental versatility or regulative capacity of ESCs and ESC-like populations that has been shown to surface in several studied species once such populations are removed from their native embryonic context and grown *in vitro* (Anlas and Trivedi, 2021; Steventon et al., 2021; Moris et al., 2020; Rossant and Tam, 2021; Dan-Sohkawa et al., 1986; Green et al., 2004; Schauer et al., 2020; Fulton et al., 2020; Williams and Solnica-Krezel, 2020). Likewise, such systems can reveal mechanisms and possibilities that will inspire a closer look at the *in vivo* setting; for example, in the context of endoderm formation, the necessity of EMT was challenged in the studies conducted in gastruloids (Vianello and Lutolf, 2020 preprint; Hashmi et al., 2022) and was further confirmed *in vivo* (Scheibner et al., 2021).

Our work aims to detail the earliest developmental events in gastruloids. The datasets generated will be of interest to other researchers investigating symmetry-breaking events as well as in determining the limitations of gastruloids to explore mouse embryonic development. Analysis of the developmental potential of ESCs aggregated and grown *in vitro* under minimal conditions can reveal previously uncharted features of early embryogenesis that cannot be elucidated from studying only the native embryo.

## MATERIALS AND METHODS

### mESC culture

Mouse (*Mus musculus*) ESCs (mESCs) were cultured as previously described (Anlas et al., 2021). In brief, Bra::GFP mESCs (Fehling et al., 2003) (obtained from Alfonso Martinez Arias, Cambridge, UK) were maintained in ES-LIF (ESL), consisting of KnockOut DMEM (ThermoFisher 10829018), 1× MEM Non-Essential Amino Acids Solution (100×, ThermoFisher 11140050), 1× GlutaMAX (100×, ThermoFisher 35050061), 1× sodium pyruvate (100×, ThermoFisher 11360070), 1× penicillin/streptomycin (100×, ThermoFisher 15140122), 50 µM β-mercaptoethanol (50 mM, ThermoFisher 31350010), 10% fetal bovine serum (FBS) (batch tested) and laboratory-made leukaemia inhibitory factor (LIF, also batch tested).

LIF-conditioned medium was generated using HEK293T cells transfected with a pCMV_LIF plasmid. Batch validation was achieved by performing the following tests with complete ESL medium containing the LIF using a E14tg2a mESC line: (1) a clonal assay and alkaline phosphatase (AP) staining; and (2) steady-state culture test for five passages and assessment for pluripotency by morphology and AP staining. A FBS batch test was performed over 4-6 different commercial batches. Validation was achieved by performing the following tests with complete ESL medium containing the respective 10% FBS to be assessed using a E14tg2a mESC line: (1) a clonal assay and alkaline phosphatase (AP) staining; and (2) a steady-state culture test for five passages and assessment for pluripotency by morphology and AP staining.

ESL preparation and LIF, as well as FBS batch testing were conducted by the Tissue Engineering Unit of the Centre for Genomic Regulation (CRG, Barcelona, Spain). In general, T::GFP mESCs were cultured on 0.1% gelatin-coated (Millipore, ES-006-B) tissue culture-treated 25 cm^2^ flasks (T25 flasks, Corning, 353108) in a humidified incubator (37°C, 5%*CO*_2_, Thermo Fisher Scientific). mESCs were passaged every 2-3 days and the ESL was exchanged for fresh, pre-warmed medium on alternating days. Cells were cultured up to 50-80% confluency before passaging or experimental use.

### Generation of gastruloids

Gastruloids were generated as previously described (Anlas et al., 2021). In summary, mESCs growing in ESL were gently rinsed with 5 ml pre-warmed DPBS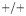 (Dulbecco's phosphate-buffered saline with *Mg* and*CaCl*_2_, Sigma, D8662). DPBS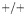 was replaced with 1 ml pre-warmed Trypsin-EDTA (Gibco, 25300) and the cells were incubated at 37°C for 1-2 min. Trypsin-EDTA was then neutralized by application of 4 ml ESL. Cells were collected via gentle pipetting, the suspension was transferred to a 15 ml centrifuge tube and centrifuged for 3 min at about 180 ***g*** (900-1000 rpm).

The supernatant was then aspirated and cells were resuspended in 10 ml warm DPBS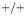 (washing). This step was repeated and, after another centrifugation round (3 min, 180 g), the pellet was resuspended in 0.5 ml to 1.5 ml of pre-warmed differentiation medium N2B27 (Ndiff227, Takara, Y40002) via pipetting up and down 5-15 times with a P1000 pipette until remaining clumps of mESCs were separated into single cells.

Hereafter, mESCs were counted using an automated cell counter (Countess II, Invitrogen). For this, 10 µl of cell suspension were mixed with 10 µl staining mix (Invitrogen, T10282) and loaded on a counting slide (Invitrogen, C10228). The calculated volume of the cell suspension was then added to the required amount of warm N2B27 and transferred to a sterile reservoir. Using a microchannel pipette, 40 µl of plating suspension were added to each well of a U-bottom, low-adhesion 96-well-plate (96WP, Greiner, 650970), which was thereafter returned to the incubator and maintained at 37°C and 5%*CO*_2_.

After 48 h, further 150 µl of pre-warmed N2B27 containing 3 µM CHIR99 (Sigma, SML1046) were pipetted into each well, followed by daily medium exchange with only N2B27 if gastruloids were grown beyond 72 hpa.

### Wide-field imaging and quantitative analysis of gastruloids

Cell aggregates cultured in round-bottom 96WPs were imaged with an Opera Phenix HSC system (PerkinElmer) in wide-field mode using a 10× air objective (NA 0.3). Focus heights were manually adjusted for a given plate, depending on size of the gastruloids. For time-lapse experiments, the incubator module was set at 37°C and 5%*CO*_2_. Medium evaporation was prevented by replacing the lid of the round-bottomed 96WP with a MicroClime environmental microplate lid (Labcyte, LLS-0310) filled up with DPBS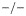 (DPBS without *Mg* and*CaCl*_2_) to maintain humidity. Images were then acquired at 10 min intervals.

After acquisition, images were compiled in single multichannel files. All images were segmented, analysed and plots were generated via MOrgAna, a machine learning (ML) pipeline implemented with a custom-written, open source Python code (Gritti et al., 2021). The segmentation was performed using only the bright-field (BF) image: 2 to 3 images per plate (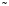
3%) were randomly chosen and a mask was manually annotated, thus generating the ground truth (GT) images. BF and GT were then used as input of the downstream ML pipeline. First, images were downsampled by a factor of two to improve computation speed.

Next, for all input BF images, ∼350 features were extracted, including difference of gaussians, gradients of gaussians, Laplace filters and DAISY descriptors with different variance values. A logistic regression model was trained to predict, for each pixel, the probability of being classified as background, inside a gastruloid or at the gastruloid edge. The trained model was thereupon used to classify the pixels in all other images in the 96WP.

Because of the variability observed in BF image quality, an additional step was implemented to ensure proper segmentation of the gastruloids. The classification output generated an image containing the identity of every pixel as well as the probability map for the ‘gastruloid edge’ class. Using the second map, watershed segmentation was used to generate an alternative segmentation. As a result of this preliminary segmentation, we hence obtained: i) a classifier mask and ii) a watershed-based mask.

Upon visual inspection, we observed that the watershed mask was accurate enough and properly segmented the gastruloid image in ∼90-95% of the cases. Otherwise, the classifier mask could be employed to yield an accurate segmentation. For the few cases in which neither watershed not classifier masks produced an acceptable segmentation, the pipeline further included the option to generate a mask manually.

The final mask was smoothened using standard morphological operations, and the area and the perimeter of the gastruloid were extracted. To account for the bending of gastruloids during elongation, eccentricity was computed on a computationally straightened version of the final mask. Briefly, we used distance transform to find the midline and the width of the gastruloid, and computed eccentricity according to 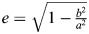; here, *a* and *b* represent the major and minor axis length of the ellipse with the same second moments as the straightened gastruloid mask.

To compute the AP fluorescence intensity profiles, for every position along the midline of the gastruloid, the average pixel intensity in the fluorescence image channel along the direction orthogonal to the midline was computed. The AP profile was then oriented such that the posterior part of the gastruloid always represented the highest T-expressing pole. For analysis across conditions, the gastruloid length was normalized to values between 0 (anterior) and 1 (posterior). T fluorescence values were normalized either globally (i.e. using all datasets included in the respective plot) or for each group/condition (i.e. separately for all replicates of a given condition in a plot including several conditions).

We remark that midline and AP fluorescence intensity profile computation may produce more variable results in gastruloids at 24 hpa, given their largely spherical shape at this developmental stage, potentially leading to arbitrary midline placement. Nevertheless, if averaged across several replicates (roughly *n*>5), we find that, for a given population, the quantifications generally correctly capture either the absence of polarization or the beginning thereof, as the presence of a discernible polarized T domain tends to be heavily associated with at least a slight ovoid shape along the thereby defined AP axis.

Quantifications of images acquired on the Opera Phenix system generally represent data from one 96WP, encompassing several conditions (up to 4) and potentially imaged at consecutive developmental days, except for Fig. 4A,B, which depicts data from two independent plates and/or experiments. Results were corroborated by performing (at least) one more experimental replicate.

### *In situ* hybridization chain reaction and image quantifications

Gastruloids were harvested from round bottom 96WPs in UV-sterilized OCPs and transferred to 2 ml Eppendorf tubes using RNase free wide-bore P1000 pipette tips. After three washes for 5 min each with 1 ml DPBS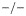, samples were fixed overnight at 4°C in 1 ml 4% PFA in DPBS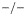. The following day, samples were dehydrated by three DPBS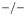 washes (5 min each, room temperature), four washes in 100% methanol (MeOH) for 10 min, each at room temperature and one wash in 100% methanol for 50 min at room temperature. Hereafter, samples were stored at -20°C, if required.

Rehydration was performed through a series of sequential washes at room temperature: 5 min in 75% methanol in DPBS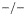 with 0.1% Tween (PBST2), 5 min in 50% methanol in PBST2, 5 min in 25% methanol in PBST2 and five times in PBST2 for 5 min each. Samples were then transferred to a 1.5 ml Eppendorf tube and 500 µl probe hybridization buffer (PHB, Molecular Instruments, HCR v3.0) was applied for 30 min at 37°C. Immediately afterwards, probe solution was prepared by adding 2pmol of each probe of interest (all probes were ordered through Molecular Instruments for HCR v3.0) into 500 µl PHB and maintained at 37°C for 30 min. Thereupon, probe solution was applied to the samples, replacing the PHB, and incubation was performed overnight at 37°C.

The next day, four washes (15 min each, 37°C) were conducted in 500 µl probe wash buffer (PWB, Molecular Instruments, HCR v3.0) preheated to 37°C and two washes (5 min each, room temperature) were performed in 5× SSCT (5× sodium chloride sodium citrate, 0.1% Tween in ultrapure*H*_2_*O*). Hereafter, amplification buffer (Molecular Instruments, HCR v3.0) was equilibrated at room temperature and SSCT was replaced with 500 µl amplification buffer (equilibrated at room temperature), followed by incubation for 30 min at room temperature.

30 pmol of each hairpin (h1 and h2, Molecular Instruments, HCR v3.0) were then separately aliquoted (2 µl of 3 µM stock), heated to 95°C for 90 s and cooled for 30 min in the dark at room temperature. Hairpins were then mixed in 500 µl amplification buffer and pre-equilibrated at room temperature to a final concentration of 60 nM per hairpin. Amplification buffer was removed from samples and replaced with hairpins in amplification buffer, followed by incubation overnight at room temperature, in the dark.

Five washes in SSCT were then performed at room temperature as follows: 2×5 min, 2×30 min and 1×5 min. Optionally, nuclear stain SiR-DNA (Spirochrome) was added with the final wash at a dilution of 1:1000. Samples were then stored at 4°C in the dark for 24 h to 2 weeks before imaging. Gastruloids were transferred to a flat-bottomed CellCarrier-96 ultra plate (PerkinElmer, 6007008) and imaged on an Opera Phenix HCS system (PerkinElmer) in confocal mode, using a 20× water objective.

For display purposes, single representative gastruloids were cropped from the original images and rotated using FIJI/ImageJ (Schindelin et al., 2012; Rueden et al., 2017). This is because samples were imaged in random orientations due to the Opera Phenix HCS not allowing manual stage movement, leading to many samples overlapping each other or being distributed along the edge of the field of view.

For multi-channel averaged AP fluorescence intensity analysis of hybridization chain reaction (HCR) datasets, a custom add-on Python script for MOrgAna was employed after generating sum intensity projections of HCR imaging data in FIJI and calculating masks, as well as AP fluorescence intensity separately for each channel as described above. Briefly, the T channel was used as the reference to delimit the AP axis, and fluorescence intensity from the other channels was plotted along this pre-determined axis. Fluorescence intensities for all channels were normalized to their own respective maxima.

### Light sheet fluorescence microscopy imaging of gastruloids

For light sheet fluorescence microscopy (LSFM) imaging, Bra::GFP gastruloids were grown in 96WP, as described above, and transferred into a Viventis LS1 live system sample holding chamber filled with 0.5 ml N2B27 containing 1× penicillin/streptomycin (Gibco, 15140122) (N2B27PS) using a P200 pipette tip cut off with sterilized scissors. 1 ml N2B27PS was then carefully added and the chamber was mounted on the LS1 live system for imaging. Imaging was performed with a Nikon 25XW NA 1.1 water immersion objective and images were captured on an Andor Zyla 4.2 sCMOS camera.

For some samples, SIR-DNA (Spirochrome, SC007) was applied at a concentration of 0.75 µM in ESL to the mESCs for at least 4 h before aggregation before gastruloid generation for light sheet imaging. This additional fluorescent channel later enabled the determination of overall sample shape during the imaging process.

### Optical flow analysis

To measure velocity fields associated with collective cellular movements, we used the signal obtained from the T::Bra GFP channel in light-sheet timelapses. The T::Bra GFP signal is present at variable levels dependent on the cell differentiation stage. We use a custom-made Matlab optic flow code based on the Kanade Lucas Tomasi (KLT) algorithm with a level 3 pyramid and a final window size of 20 pixels (square of 1 cell by 1 cell) (Lucas and Kanade, 1981) with a measurement every 10 pixels. The timelapses were registered (rigid transformation, via StackReg in FIJI/ImageJ) before computing the velocity field. Owing to the fluctuating nature of the flow, we time-averaged the velocity field over 16 consecutive frames and graphically represented it every four frames.

### Immunohistochemistry

For fixation, aggregates were harvested using organoid collection plates (OCPs, patent EP3404092A1) and subsequently pooled in 2 ml Eppendorf tubes with a cut-off P1000 pipette tip. After three washes for 5 min each with 1 ml DPBS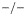 (DPBS without *Mg* and*CaCl*_2_, Sigma, D8537), 1 ml 4% paraformaldehyde (PFA) in DPBS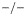 was applied and samples were incubated for 2 h at 4°C with gentle horizontal rotation. Hereafter, gastruloids were washed three to five times with 1 ml DPBS^−/−^ each. At this point, aggregates were optionally stored at 4°C for a few days to 1 week.

All subsequent steps were conducted at 4°C and under gentle shaking, if not stated otherwise. Aggregates were washed three times for 10 min with 500 µl DPBS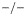 containing 9% foetal bovine serum (FBS), 1% BSA (Jackson Immuno Research, 001-000-162) and 0.2% Triton X-100 (PBSFBT). For blocking, gastruloids were incubated for 2 h in PBSFT on an orbital shaker. In case of single-round antibody (AB) stainings, anti-GFP-AF647 (ThermoFisher, A-31852) and phalloidin AF555 (ThermoFisher, A34055) were applied at 1:500 in 500 µl PBSFBT overnight.

The next day, samples were washed with PBSFBT three times for 30 min. Afterwards, aggregates were washed three times with DPBS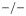 containing 0.2% FBS and 0.2% Tween (PBT) for 15 min each. Gastruloids were then removed from the orbital shaker and PBT was replaced with 100 µl Vectashield with DAPI (Vector Laboratories, H-1200-10) per 2 ml tube. Using a cut-off P200 tip, 10-20 gastruloids per condition were then transferred into a well of a flat-bottomed CellCarrier-96 ultra plate (PerkinElmer, 6007008) and imaged on an Opera Phenix HCS system (PerkinElmer) in the confocal mode, using a 20× water objective lens.

For image display purposes, representative gastruloids were cropped from the original images and rotated using FIJI/ImageJ (Schindelin et al., 2012; Rueden et al., 2017). This is because samples were imaged in random orientations due to the Opera Phenix HCS not allowing manual stage movement, leading to many samples overlapping with each other or being distributed along the edge of the field of view.

### Preparation of gastruloids for scRNA-seq

To ensure sufficient numbers of dissociated cells for each experimental condition, five 96WPs of gastruloids were generated for the 24 h timepoint, three for the 48 h timepoint and two for the 72 h timepoint. For the 0 h timepoint, T::GFP mESC were grown in a T25 flask as described above. Individual plates were harvested using UV-sterilized OCPs and gastruloids were pooled into a 15 ml centrifuge tube using RNase-free wide-bore P1000 pipette tips (Thermo Fisher Scientific, 2079G). When all plates from a given condition were harvested, the centrifuge tube was placed into an incubator (37°C, 5%*CO*_2_) and harvesting proceeded with plates from the next condition.

Once all samples were collected, thus yielding one 15 ml centrifuge tube per condition, aggregates were centrifuged at 180 ***g*** for 2 min. The supernatant was then removed, 1 ml TrypLE (Thermo Fisher Scientific, 12604) was added per tube for dissociation and samples were incubated for 5 min at 37°C (5%*CO*_2_). TrypLE was deactivated using 4 ml ESL and gastruloids were centrifuged for 2 min at 180 ***g***. Supernatant was removed, samples were washed with 5 ml ice-cold (4°C) 0.1% RNase-free BSA (Thermo Fisher Scientific, AM2616) in DPBS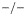 (BPBS) and centrifuged (2 min at 180 ***g***). From this step onwards, samples were maintained at 4°C.

After removal of the supernatant, the pellet was resuspended in 50-100 µl ice-cold BPBS (depending on pellet size) by gently pipetting up and down two or three times with a P200-P1000 tip. The cell suspension was then filtered through a 35 µm filtered cap tube (Corning, 352235). Cells were counted as previously described, aiming for a concentration of around 1×10^6^ cells/ml. In case of high concentrations (>1.5×10^6^ cells/ml), the cell suspension was diluted with ice-cold BPBS and re-counted.

For the 0 h timepoint, mESCs were trypsinized using 1 ml Trypsin-EDTA 0.05% as described above. After neutralization (4 ml ESL) and centrifugation (180 ***g***, 3 min), the supernatant was discarded and cells were washed with 5 ml ice-cold BPBS. Cells were then kept on ice, until samples from other experimental conditions reached this washing step. At this point, all samples were joined together for centrifugation and further procedures illustrated above.

### Library preparation and sequencing

After the generation of single-cell suspensions in BPBS as described above, cells in each sample were barcoded with a Chromium Single Cell 3′ GEM, a Library & Gel Bead Kit v3 (10x Genomics) and a Chromium Single Cell B Chip Kit (10x Genomics) on a Chromium Controller (10x Genomics), followed by cDNA library construction according to manufacturer's instructions. These libraries were sequenced using a NextSeq 500 system (Illumina). We read 8, 28 and 56 bp for Tru-seq indices, 10x barcodes with unique molecular identifiers (UMIs) and fragmented cDNA, respectively.

### Basic scRNA-seq data analysis

Quality control (QC), alignment to the mouse genome (GRCm38) and counting of the sequence reads were conducted with CellRanger v3.1.0 (10x Genomics) to generate feature-barcode matrices. The summary of the statistics of sequencing results is shown in Table S1.

Further QC steps, normalization, identification of most variable features (*n*=3000), scaling, PCA and clustering were performed with Seurat version 3.2.2 (Stuart et al., 2019) loaded into R version 4.0.3. Low-quality cells were removed with the following thresholds: unique feature count (UFC) >2500 & mitochondrial count (MC) <10% & total RNA count (TRC) <150,000 for the 0 h dataset; UFC > 2750 & MC < 15% & MC > 1% & TRC < 100,000 for the 1st replicate of the 24 h dataset; UFC > 3750 & MC < 15% & MC > 1% & TRC < 150,000 for the 2nd replicate of the 24 h dataset; UFC > 2750 & MC < 15% & MC > 1% & TRC < 100,000 for the 1st replicate of the 48 h dataset; UFC > 3500 & MC < 15% & MC > 1% & TRC < 120,000 for the 2nd replicate of the 48 h dataset; and UFC > 2500 & MC < 20% & MC > 1% & TRC < 135,000 for the 72 h dataset.

Experimental replicates of 24 and 48 hpa datasets were separately integrated using the ‘FindIntegrationAnchors()’ and ‘IntegrateData()’ functions (with dims=1:50) of Seurat in R. All datasets (the integrated 24 hpa and 48 hpa datasets as well as the 72 hpa dataset) were then integrated as described above. PCA analysis and UMAP clustering was then performed using the ‘RunPCA()’, ‘FindNeighbors()’, ‘FindClusters()’ and ‘RunUMAP()’ functions (with dims=1:50). Cell cycle bias on clustering was assessed via the ‘CellCycleScoring()’ function and manual inspection of most differentially expressed genes for each cluster. We did not opt for cell cycle correction, as clustering results did not appear skewed by such cell cycle factors. For the UMAP plots, gene expression information was pulled from the ‘RNA’ assay slot.

Most differentially expressed cluster marker genes were identified through the ‘FindAllMarkers(..., min.pct=0.25, logfc.threshold=0.25)’ function in the ‘RNA’ assay slot. GO-term analyses were performed using ‘ClusterProfiler’. For comparing expression of selected germ layer genes between all timepoints, datasets were merged with the ‘merge()’ function of Seurat.

In order to analyse and compare T^+^  with T^−^  populations across timepoints, cells from each dataset were separated based on normalized expression level cut-offs (using the ‘RNA’ assay slot) of >1.5=T^+^  and ≤0.05=T^−^. In turn, for comparing Sox2^+^  with Sox2^−^  population, cut-offs were >1.0=Sox2^+^  and ≤0.05=Sox2^−^. Differentially expressed genes and GO-term analysis was then performed as described above.

Integration of the 0 h T::GFP mESC data from this study with a E14 wild-type mESC dataset (Alda-Catalinas et al., 2020) was conducted via the ‘fastMNN’ function in R using a list of the 3000 most variable genes filtered for common cell cycle markers. UMAP analysis and clustering were performed using the first 25 PCs.

### RNA velocity analysis

The scRNA sequencing datasets of gastruloid cells from 24, 48 and 72 hpa were used to perform RNA velocity analysis. To achieve this, we used one replicate and first generated an annotated loom file using the velocyto Python package (La Manno et al., 2018) and the genome annotation file from GENCODE. Next, RNA velocity was performed using scVelo in ‘stochastic’ mode (Bergen et al., 2020). The resulting embedding streams were overlaid on the integrated UMAP plot and cells were color-coded according to their cluster identity.

### Pseudotime analysis

For pseudotime analysis, we used the Monocle3 toolkit (version 1.3.1) (Qiu et al., 2017; Cao et al., 2019) loaded into R (version 4.2.2). In brief, the seurat object of our integrated sc-dataset (24, 48 and 72 hpa) was converted into a ‘cell data set’, which was then annotated with the corresponding gene name, expression and cluster (partition) data from the original seurat object. We then built trajectories using the ‘learn_graph()’ function (in this case, setting the ‘use_partition’ argument to ‘TRUE’ or ‘FALSE’ did not make a difference) and then ordered cells in pseudotime via the ‘order_cells()’ function with the pluripotent I cluster designated as the root cell population. The generated pseudotime values were then transferred back into the seurat object for line-plot generation via ggplot2 (version 3.4.2). Expression curves for genes of interests were generated – based on the normalized expression value (for the respective gene) of individual cells ordered via their assigned pseudotime value – with the ‘geom_smooth’ function, using the ‘gam’ method and the formula 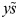
(*x*, *bs* = “*cs*”).

### Integration of gastruloid and mouse scRNA datasets

For label transfer analysis, for every gastruloid cell, we identified the closest mouse cell within the reference atlas (Pijuan-Sala et al., 2019) in 50-dimensional PCA space using the NearestNeighbors algorithm, and assigned the mouse cell label. The correlation heatmap was performed by computing the Pearson correlation coefficient between the average values of the PCA components for each cluster pair. A major discrepancy was found whereby the majority of gastruloid neural-like cells mapped to mouse mesodermal cells, despite showing highest correlation with the mouse ectoderm cluster based on principal components (PCs, Fig. S9D).

This prompted us to re-evaluate the label transfer process by computing different quality control (QC) parameters as follows.

(1) PC-space distances (averaged for each gastruloid cluster) between each gastruloid cell to the 30 closest mouse cells (Fig. S9E). Cell distances were computed as the Euclidean distance between each gastruloid cell and mapped mouse cell. Well integrated cells, such as cells within the primitive streak cluster, show short distances to their mapped mouse cells.

(2) The labelling agreement or score (Fig. S9F), referring to the fraction of those 30 cells that correspond to the actually assigned label (i.e. the label of the closest cell).

(3) The percentage of unique mapped cells (Fig. S9G), which represents the number of unique mouse cells to which gastruloids cells mapped, divided by the total number of gastruloid cells within that cluster. This number is close to 1 for ‘well-mixed’ cells, for which the probability of two gastruloid cells mapping onto the same mouse cell is negligible.

Upon inspection, these parameters reveal probable sources of the inconsistencies seen in the UMAP, but particularly that gastruloid cells in many clusters (pluripotent, primed pluripotent, early differentiated, mesodermal I and neural-like) map to relatively few unique mouse cells (unique mapping percentages <50%).

Gastruloid scRNAseq data obtained at 24, 48 and 72 hpa were thus integrated to the aforementioned comprehensive reference dataset (Pijuan-Sala et al., 2019) using mutual nearest neighbour as previously described (Argelaguet et al., 2019). Briefly, datasets were normalized and the counts of the highly variable genes were used for principal component analysis (number of PCs=50). All the downstream analysis was performed in PC space. First, batch correction was performed on the concatenated datasets using the R function ‘reducedMNN’ from the ‘batchelor’ package. The corrected dataset of the 50 PCs was then used as input of the ‘umap’ R function (*n*_*n*_*eighbors*=20,*min*_*d*_*ist*=0.7). Cell clusters from mouse and gastruloids were redefined to consider the major germ layer lineages and individual cells were plotted on the common UMAP space and color-coded according to cluster identity or developmental stage. Integration with the other mouse atlas (Nowotschin et al., 2019), filtered to include only developmental stages E3.5-5.5, was also conducted as described above.

Differential expression analysis was performed considering pairs of cell clusters. Counts were log-normalized and the average expression within each cluster was computed. Next, highly differentially expressed genes were identified using a differential cut-off of +−1. In the figures, only the top 10 differentially expressed genes are represented.

## Supplementary Material



10.1242/develop.202171_sup1 Supplementary information

Table S1. Quality control parameters. scRNA-seq quality control parameters for all datasets generated for this study.

Table S2. Marker genes genes for clusters in the integrated dataset. List of the most differentially expressed genes for each cluster in the integrated scRNA-seq dataset (24hpa,48hpa,72hpa), identified v ia t he “ FindAllMarkers()” function in Seurat.

Table S3. Marker genes for clusters in the 72hpa dataset. List of the most differentially expressed genes for each cluster in only the 72hpa scRNA-seq dataset, identified v ia t he “ FindAllMarkers()” f unction i n Seurat.

Table S4. Summary of GO-term analysis results for the 24hpa T^-^ population. Summary of GO-term analysis results computed via the “ClusterProfiler” “enrichGO()” function.

Table S5. Summary of GO-term analysis results for the 24hpa T^+^ population. Summary of GO-term analysis results computed via the “ClusterProfiler” “enrichGO()” function.

Table S6. Summary of GO-term analysis results for the 48hpa T^-^ population. Summary of GO-term analysis results computed via the “ClusterProfiler” “enrichGO()” function.

Table S7. Summary of GO-term analysis results for the 48hpa T^+^ population. Summary of GO-term analysis results computed via the “ClusterProfiler” “enrichGO()” function.

Table S8. Summary of GO-term analysis results for the 72hpa T^-^ population. Summary of GO-term analysis results computed via the “ClusterProfiler” “enrichGO()” function.

Table S9. Summary of GO-term analysis results for the 72hpa T^+^ population. Summary of GO-term analysis results computed via the “ClusterProfiler” “enrichGO()” function.

Table S10. Summary of GO-term analysis results for the 24hpa Sox^-^ population. Summary of GO-term analysis results computed via the “ClusterProfiler” “enrichGO()” function.

Table S11. Summary of GO-term analysis results for the 24hpa Sox2^+^ population. Summary of GO-term analysis results computed via the “ClusterProfiler” “enrichGO()” function.

Table S12. Summary of GO-term analysis results for the 48hpa Sox2^-^ population. Summary of GO-term analysis results computed via the “ClusterProfiler” “enrichGO()” function.

Table S13. Summary of GO-term analysis results for the 48hpa Sox2^+^ population. Summary of GO-term analysis results computed via the “ClusterProfiler” “enrichGO()” function.

Table S14. Summary of GO-term analysis results for the 72hpa Sox2^-^ population. Summary of GO-term analysis results computed via the “ClusterProfiler” “enrichGO” function.

Table S15. Summary of GO-term analysis results for the 72hpa Sox2^+^ population. Summary of GO-term analysis results computed via the “ClusterProfiler” “enrichGO()” function.
