# Supplementary material for: Early autonomous patterning of the anteroposterior axis in gastruloids
Source: Development. 2024 Nov 18;151(22):dev202171. doi: 10.1242/dev.202171 (PMC11607688; doi:10.1242/dev.202171)
Supplement: Supplementary information [file develop-151-202171-s1.pdf]

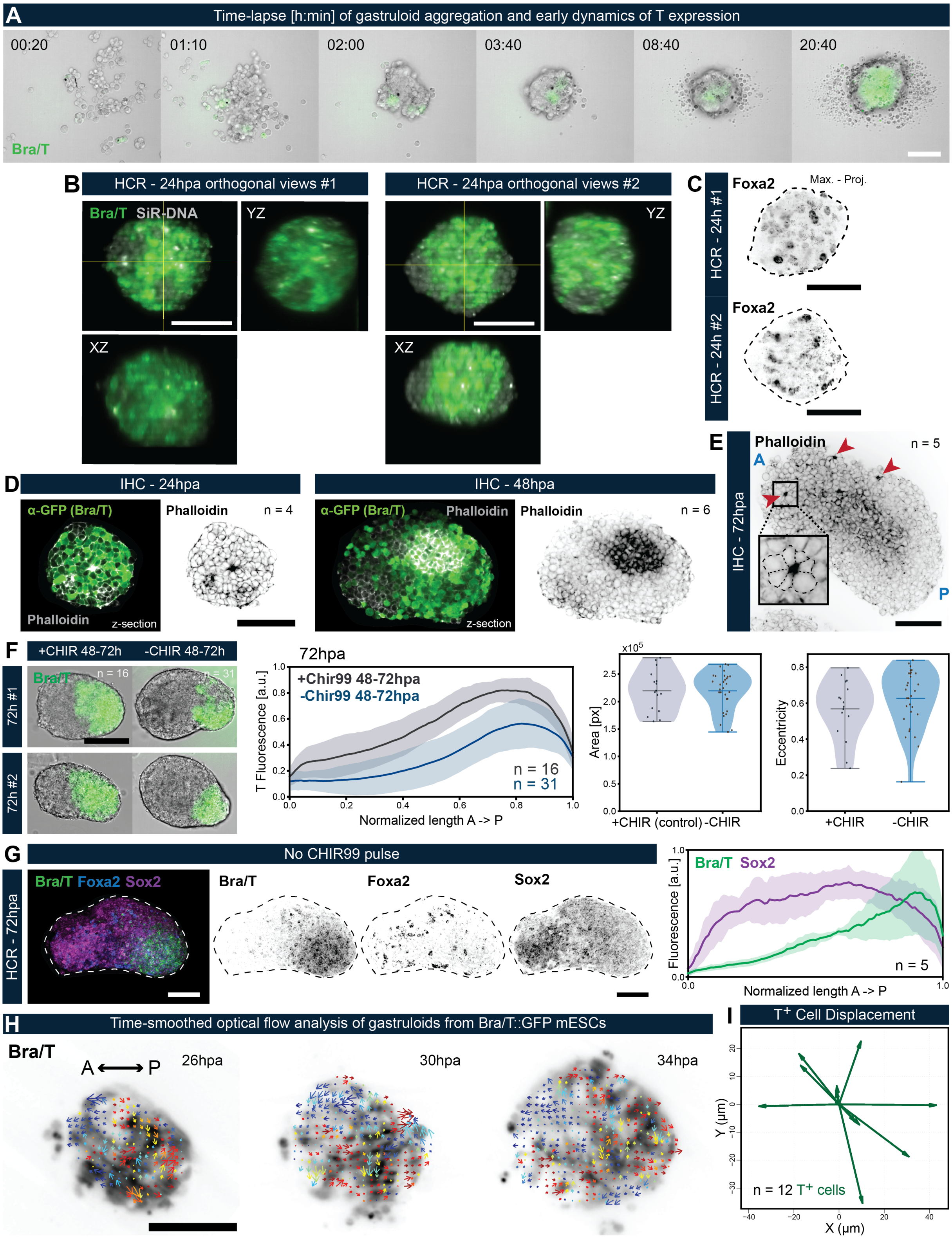

Development • Supplementary information

Fig. S1. Self-organized AP axis formation in gastruloids demarcated by T polarization (**A**) Representative images from a time-lapse of the first 24h of gastruloid development following aggregation show T activity in a subset of initial mESCs and subsequent expression dynamics. Scale bar = 100 µm. (**B**) HCR for T in gastruloids at 24hp with orthogonal views demonstrates that expression is evenly distributed along the thickness of the aggregate. Scale bars = 100 µm. (**C**) HCR for Foxa2 illustrates dispersed, but evenly distributed expression in gastruloids at 24hp, prior to T symmetry breaking. Scale bar = 100 µm.(**D,E**) IHC for Phalloidin in gastruloids at 24hp, 48hp and 72hp indicates no clear differences in cell shape between T^+ and T^- cells. However, there is occasional multicellular rosette formation in the anterior (A) region (red arrows) of gastruloids at 72hpa. Scale bars = 100 µm. (**F**) Representative images of gastruloids grown with or without canonical Wnt upregulation (CHIR99 pulse) as well as quantifications of T fluorescence intensity along the AP axis and shape descriptors (aggregate area and eccentricity). Scale bar = 200 µm. (**G**) HCR for T, Foxa2 and Sox2 in gastruloids at 72hpa not subjected to a CHIR99 pulse and quantification of fluorescence intensity along the AP axis across replicates for T and Sox2 (right panel). Scale bars = 100 µm. (**H**) Representative images of optical flow analysis of gastruloids from T::mESCs imaged via LSFM (light sheet fluorescence microscopy) between 24-40hpa. Velocity fields are overlayed on the T channel fluorescence. Arrows are color coded by directionality. Scale bar = 100 µm. (**I**) Manually tracked displacement (end-to-end distance) of 12 randomly picked T^+ cells from a representative LSFM dataset of a polarizing gastruloid imaged between 24-48hpa (out of 4 total datasets). Cells were tracked within a (roughly) 2h window. The original position of a given cell at timepoint = $t_{initial}$ was set to 0,0.

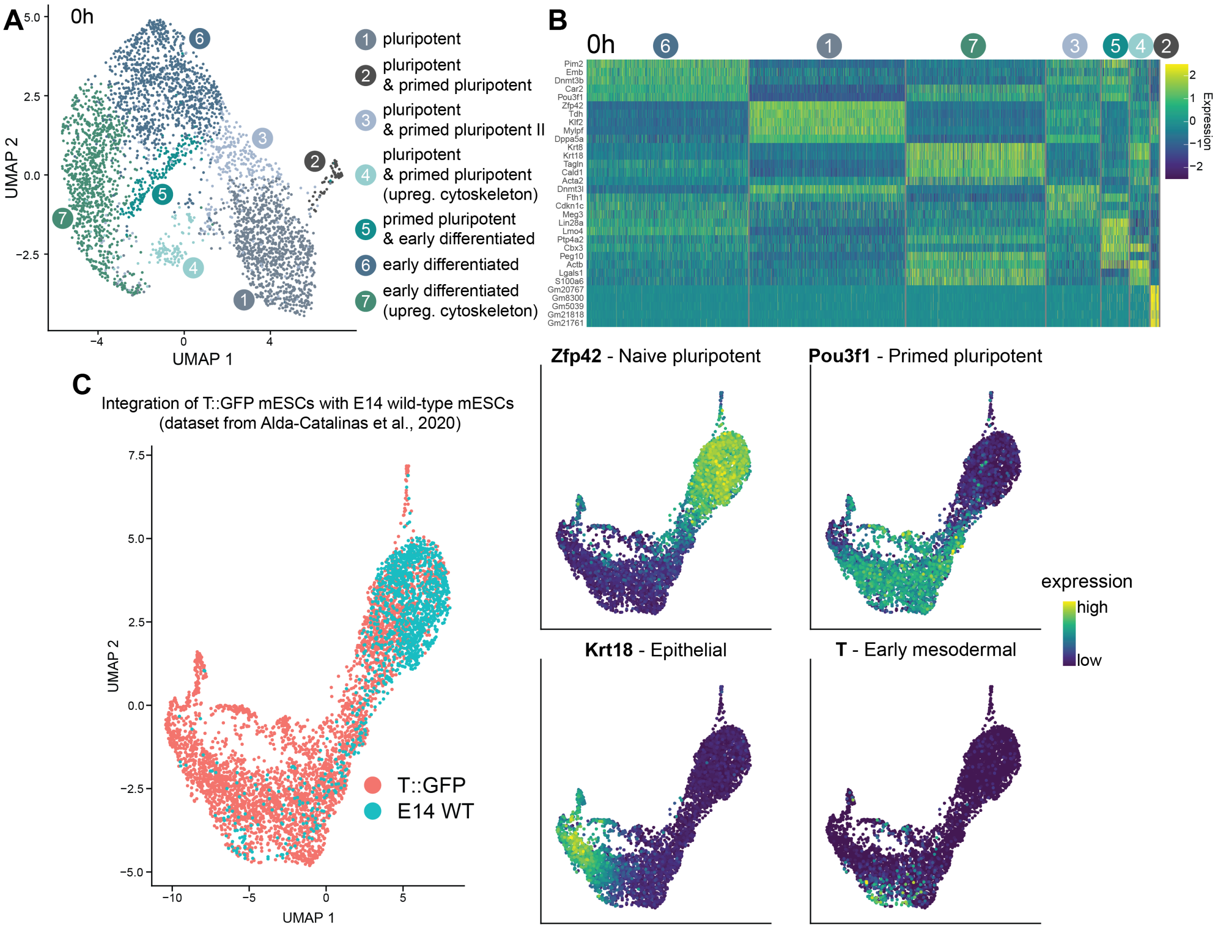

Fig. S2. Single-cell transcriptomic analysis of early gastruloid development (**A**) UMAP for mESCs prior to gastruloid formation (0hpa timepoint) with cluster annotations. (**B**) Heatmap with top 5 differentially expressed genes distinguishing 0hpa clusters. (**C**) Integration of our 0h scRNAseq data (T::GFP mESCs) with a sc-dataset of E14 wild-type mESCs from Alda-Catalinas et al.(2020) (see main text) and gene expression analysis of cell state markers (right panel) on the integrated UMAP highlighting that the T::GFPs employed in this study feature comparatively increased admixture of primed pluripotent and early differentiated populations.

Development • Supplementary information

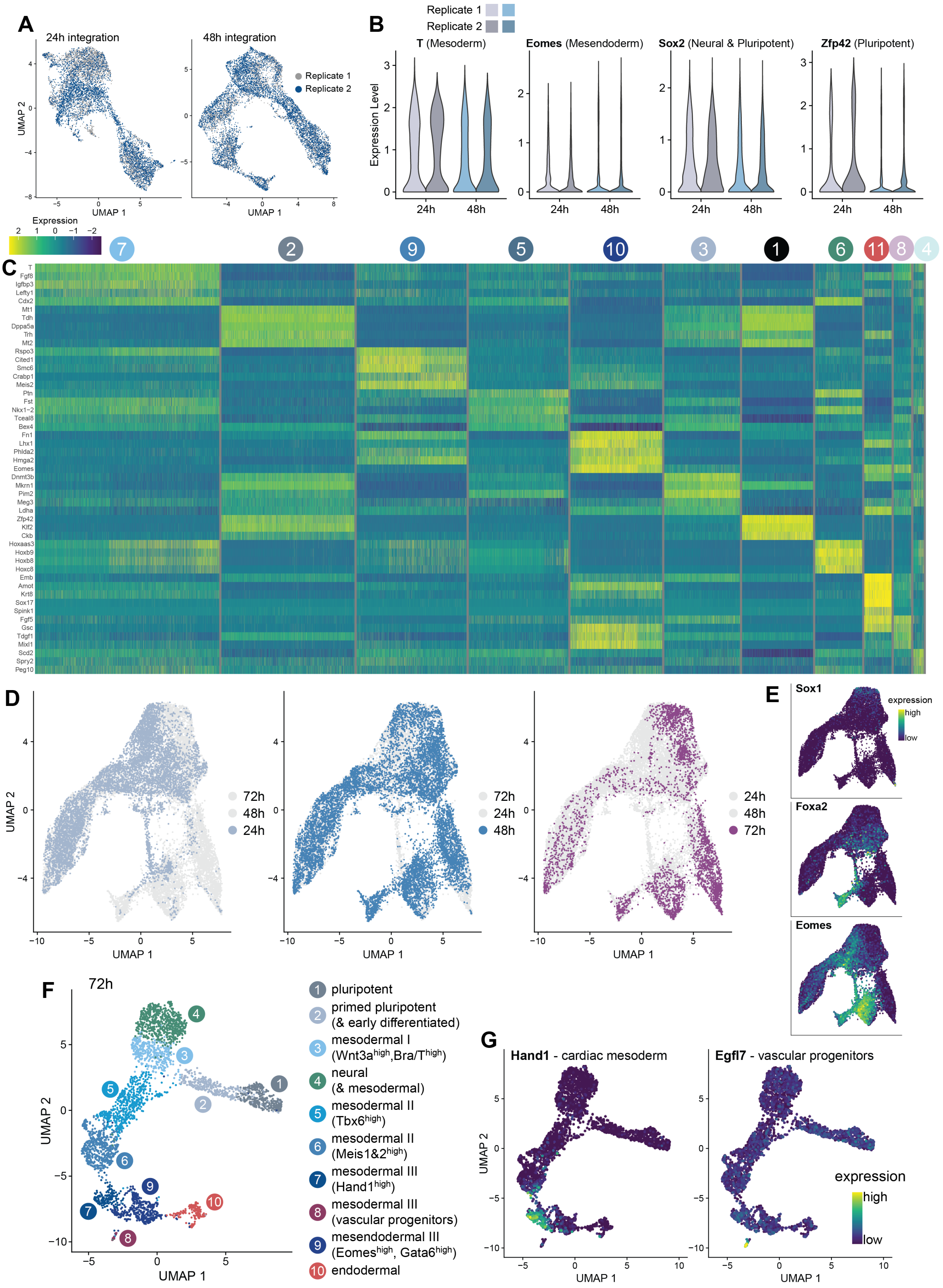
A
24h integration
48h integration
Replicate 1
Replicate 2
UMAP 1
UMAP 2
B
Replicate 1
Replicate 2
T (Mesoderm)
Eomes (Mesendoderm)
Sox2 (Neural & Pluripotent)
Zfp42 (Pluripotent)
Expression Level
24h
48h
Expression
C
7
2
9
5
10
3
1
6
11
8
4
T
Fgf8
Igfbp3
Lefty1
Cdx2
Mt1
Tdh
Dppa5a
Trh
Mt2
Rspo3
Cited1
Smc6
Crabp1
Meis2
Ptn
Fst
Nkx1−2
Tceal8
Bex4
Fn1
Lhx1
Phlda2
Hmga2
Eomes
Dnmt3b
Mkrn1
Pim2
Meg3
Ldha
Zfp42
Klf2
Ckb
Hoxaas3
Hoxb9
Hoxb8
Hoxc8
Emb
Amot
Krt8
Sox17
Spink1
Fgf5
Gsc
Tdgf1
Mixl1
Scd2
Spry2
Peg10
D
72h
48h
24h
UMAP 1
UMAP 2
E
Sox1
expression
high
low
Foxa2
Eomes
F
72h
pluripotent
primed pluripotent (& early differentiated)
mesodermal I (Wnt3a^high, Bra/T^high)
neural (& mesodermal)
mesodermal II (Tbx6^high)
mesodermal II (Meis1&2^high)
mesodermal III (Hand1^high)
mesodermal III (vascular progenitors)
mesendodermal III (Eomes^high, Gata6^high)
endodermal
G
Hand1 - cardiac mesoderm
Egfl7 - vascular progenitors
expression
high
low

Fig. S3. Single-cell transcriptomic analysis of early gastruloid development (**A**) UMAP plot validating integration quality for replicates of 24hpa and 48hpa datasets. (**B**) Comparison of expression of key germ layer markers between replicates of 24hpa and 48hpa datasets. (**C**) Heatmap featuring top 5 differentially expressed genes for each cluster of the integrated gastruloid datasets (24hpa, 48hpa and 72hpa). Cluster labels are from main text Fig. 2. (**D**) UMAPs of the integrated gastruloid dataset with cells highlighted by developmental timepoint (hpa). (**E**) Expression of neural (Sox1) as well as anterior mesoderm and endodermal markers Foxa2 and Eomes in the integrated dataset. (**F**) UMAP plot with cluster annotations for the individual 72hpa dataset highlights emergence of cardiac and vascular progenitor clusters. (**G**) Expression of markers for cardiac mesoderm (Hand1) and vascular progenitors (Egfl7) highlighted on 72hpa UMAP.

Development • Supplementary information

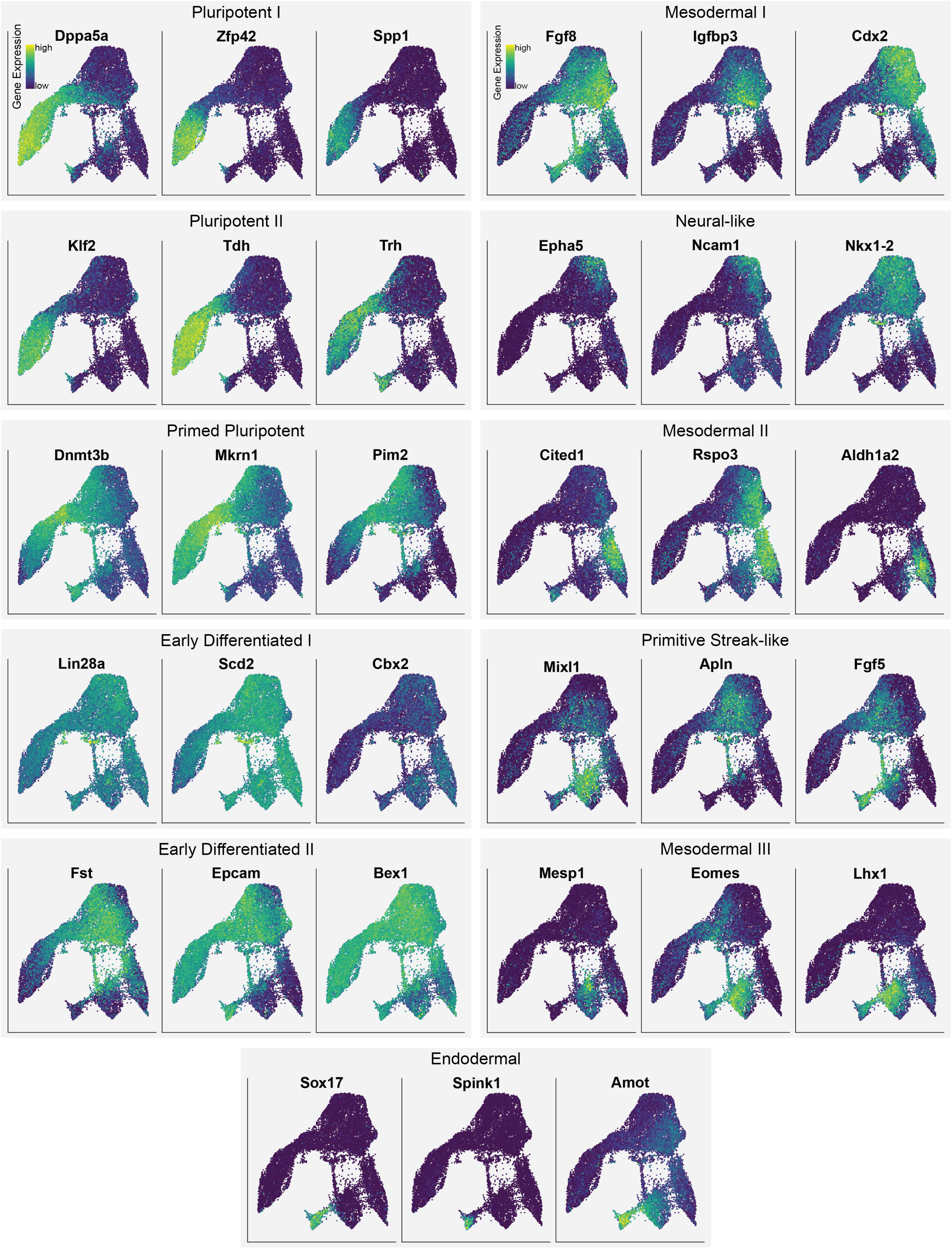

Fig. S4. Overview of UMAP cluster marker genes for integrated gastruloid dataset (24, 48, 72hpa)
Expression levels for 3 highly variable genes for each of the clusters (see main text Fig. 2) are shown on the integrated UMAP. Cluster marker genes were computed and ranked via the "FindAllMarkers" function in Seurat (see Supplementary Table 2).

Development • Supplementary information

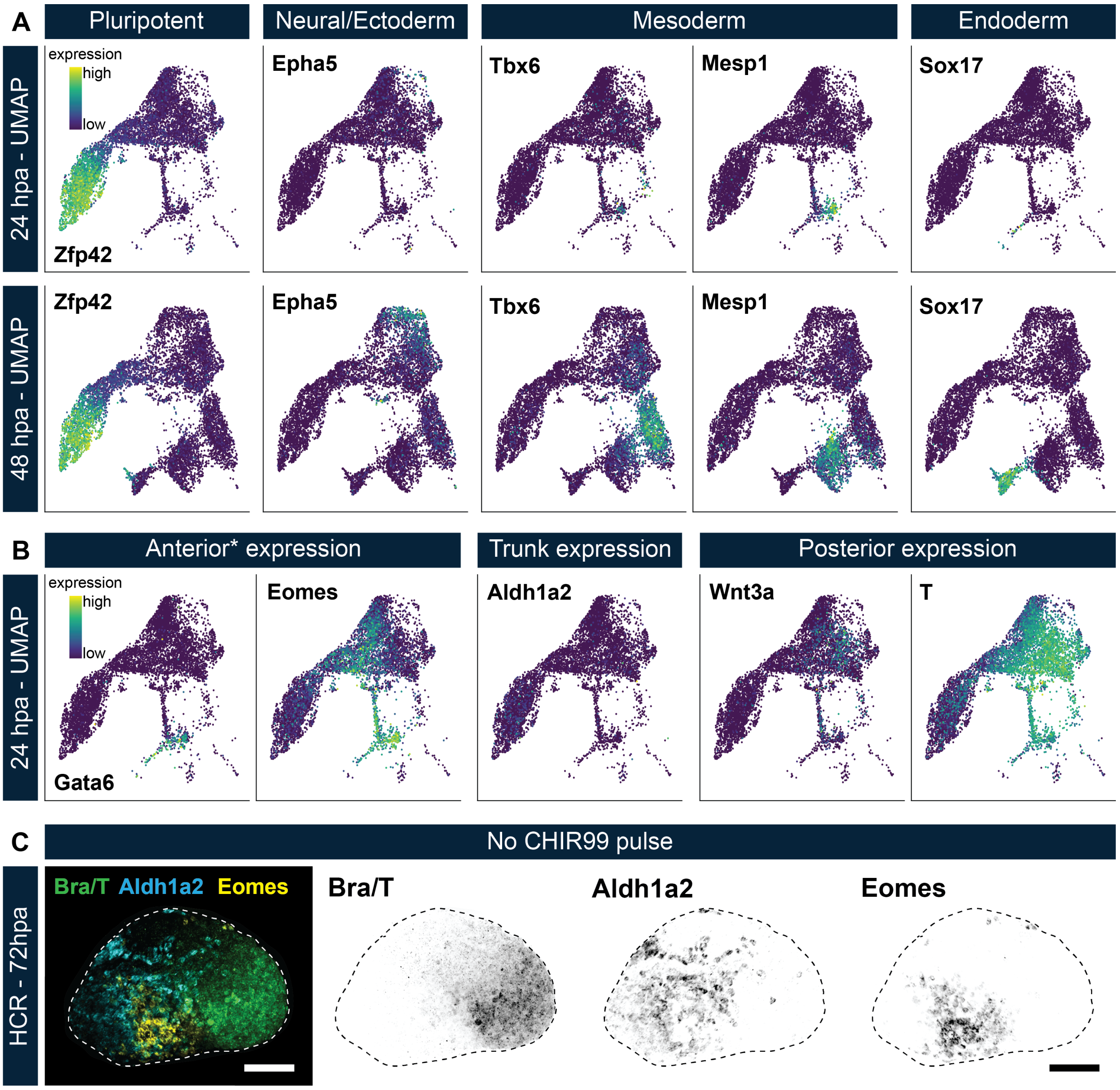

Fig. S5. AP axial patterning occurs in presence or absence of external Wnt upregulation

(**A**) Expression of germ layer marker genes Zfp42 (pluripotent), Epha5 (neural/ ectoderm), Tbx6 (paraxial mesoderm), Mesp1 (cardiac and general anterior mesoderm) and Sox17 (endoderm) in the integrated UMAP filtered for cells from the 24 and 48hpa timepoints, respectively. (**B**) Key AP axial markers Gata6 and Eomes (anterior in gastruloids), Aldh1a2 (trunk) as well as Wnt3a and T (posterior) were plotted on the UMAP of the integrated dataset filtered for cells only from the 24hpa timepoint. (**C**) Representative image for HCR stainings of gastruloids at 72hpa that were not subjected to external Wnt upregulation via CHIR99. Note how spatial segregation of T, Aldh1a2 and Eomes expression domains along the AP axis is nevertheless achieved. Scale bar = 100 µm. Fluorescent channel contrasts have been adjusted for display.

Development • Supplementary information

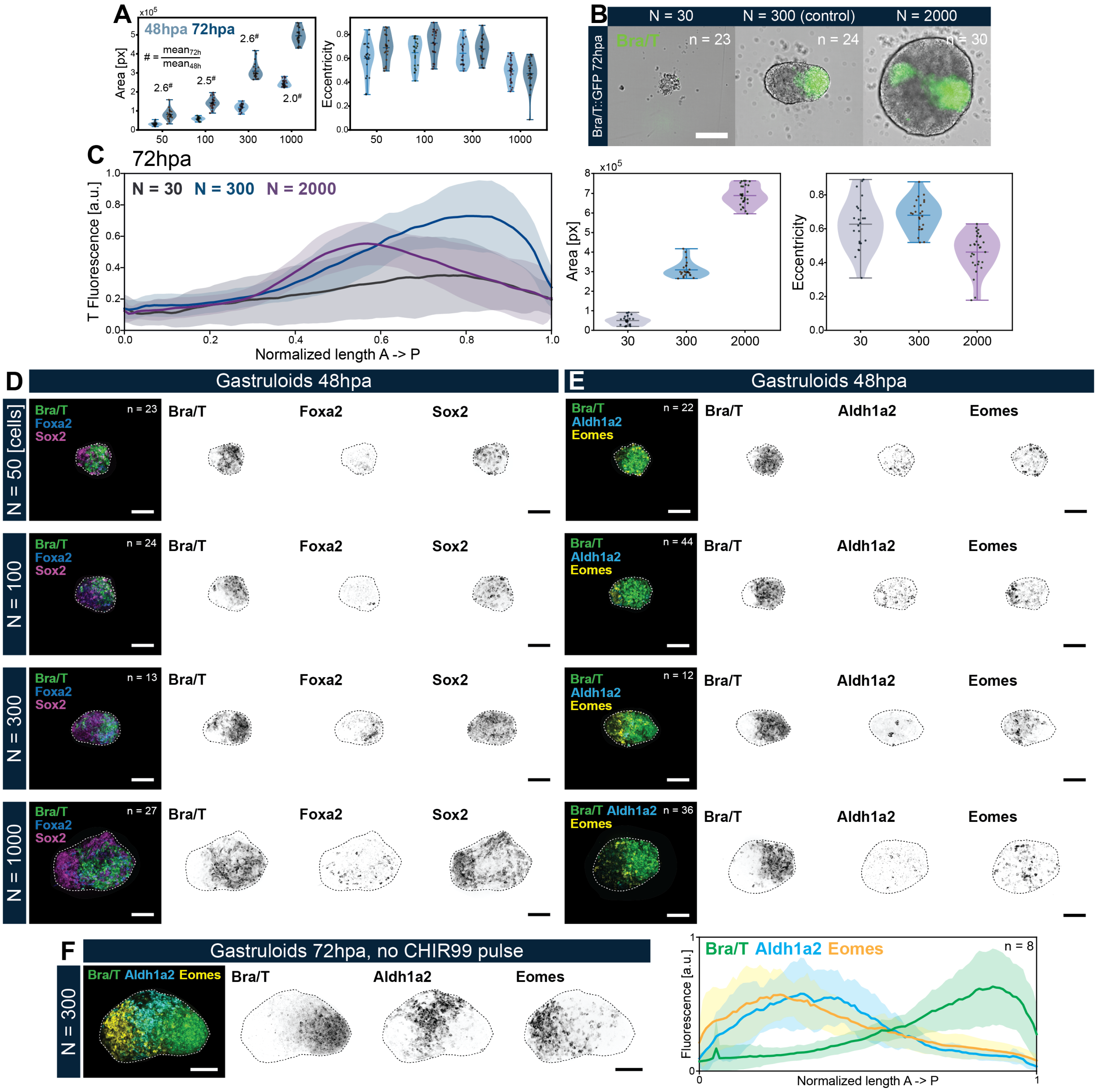

Fig. S6. T polarization and AP axial patterning is robust to changes in aggregate size

(**A**) Quantitative of shape descriptors (area and eccentricity) of gastruloids grown from differentinitial cell (N) numbers demonstrates robustness of T symmetry breaking. Note how the relative aggregate growth between 48-72hpa is similar across N. **(B,C)** Exemplary images and quantification of T fluorescence intensity along the AP axis as well as aggregate area and eccentricity for gastruloids grown from different starting cell (N) numbers demonstrating the size or cell number upper and lower limits for reproducible T polarization. Scale bars = 200 µm. **(D,E)** HCR in situs of gastruloids from different initial cell numbers at 48hpa for key germ layer markers and AP axial patterning genes T, Foxa2 and Sox2 (D) as well as T, Aldh1a2 and Eomes (E). Scale bars = 100 µm. **(F)** Representative image for HCR stainings of gastruloids at 72hpa that were not subjected to external Wnt upregulation via CHIR99 (also see Supp. Fig. 5C) and quantification of fluorescent channel intensities along the aggregate AP axis across replicates (right panel). Scale bar = 100 µm. Fluorescent channel contrasts have been adjusted for display.

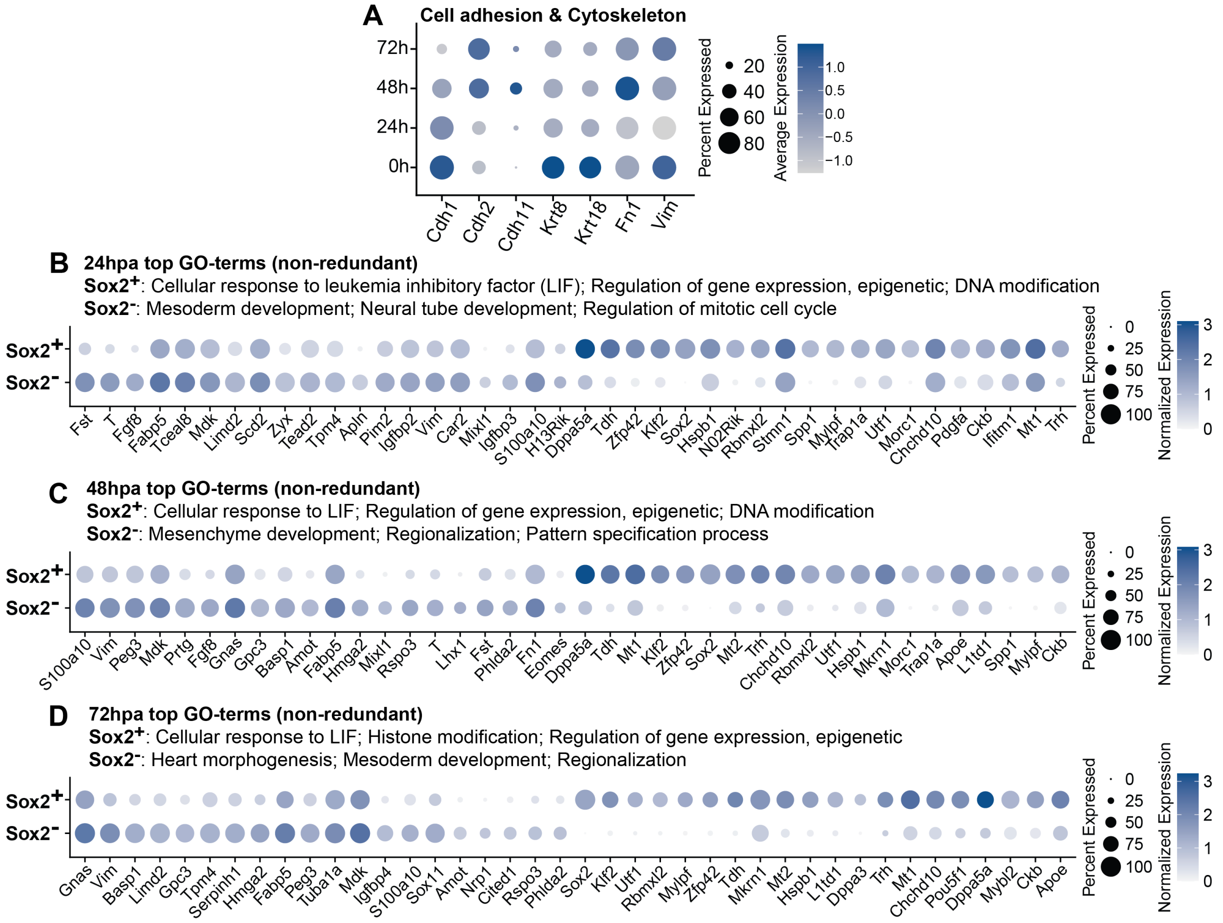

Fig. S7. Transcriptional properties of Sox2^{+} versus Sox2^{-} populations complement T^{+} versus T^{-} comparisons

(**A**) Expression of cell adhesion genes for each developmental time point. Size of circles denotes fraction of expressing cells and the color code indicates averaged expression level for a given gene. (**B-D**) Single cell transcriptomic comparison of Sox2^{+} with Sox2^{-} cell populations around the T symmetry breaking event at 48hpa. Top 3 non-redundant GO terms as well as top 20 most differentially expressed genes are shown for each population across 3 timepoints (24hpa, 48hpa, 72hpa). For each gene, the size of the circle denotes the fraction of expressing cells, the color code denotes the (normalized) expression level in those cells.

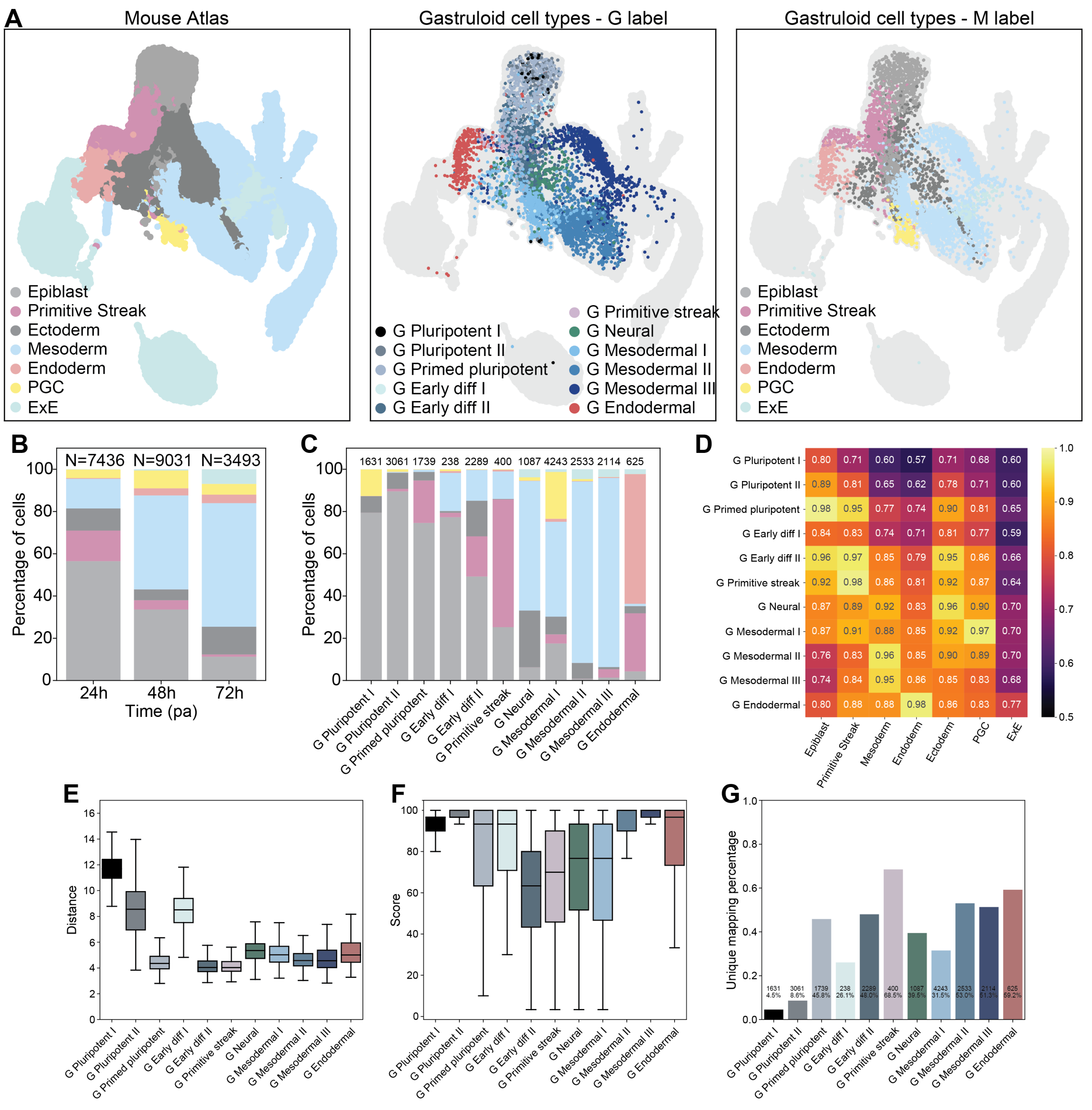

Fig. S8. Comparing germ layer formation in gastruloids to the mouse embryo (**A**) Visualization of cell label transfer of gastruloid integrated dataset (24hpa, 48hpa, 72hpa) to an estab-lished mouse sc-atlas (Pijuan-Sala et al., 2019) via UMAP. (**B**,**C**) Lineage progression of developmental time (hpa) and mouse cell type mapping quantifications for each gastruloid cluster. (**D**) Heatmap showing Pearson correlation coeÿcient between the average values of the PCA components for mouse and gastru-loid cluster pairs. Note that this is based on the 50-dimensional PC space employed for the label transfer analysis. (**E**)-(**G**) Quality control parameters for label transfer reveal several discrepancies especially for more undifferentiated populations.

Development • Supplementary information

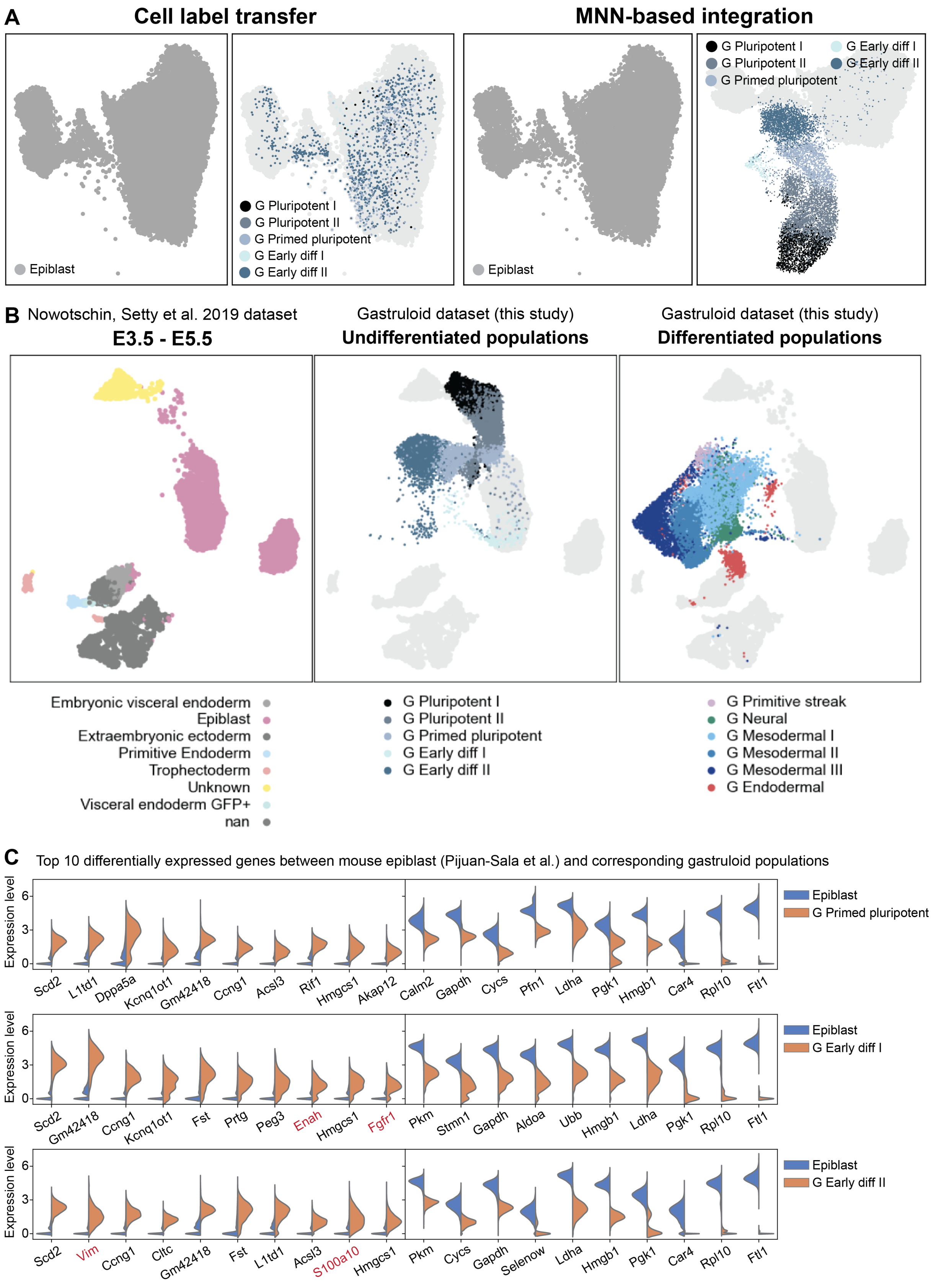

Development • Supplementary information

Fig. S9. Uncommitted cell fates in gastruloids map poorly to the mouse epi-blast

(**A**, left panel) UMAP visualization of cell label transfer of gastruloid uncommitted (pluripotent, primed pluripotent, early differentiated) cell types to the corresponding mouse epiblast population from Pijuan-Sala et al. (2019). Datasets were filtered to only include these populations. Poor MNN-based mapping between epiblast and corresponding gastruloid clusters as shown in main text Fig. 6A,B is retained even upon exclusively filtering the gastruloid and the mouse dataset for such uncommitted cell fates (A, right panel). **(B)** MNN-based integration with another mouse sc-atlas (Nowotschin et al., 2019) spanning earlier mouse development (E3.5-5.5) and hence more naive pluripotent cell populations still yields relatively poor map-ping with respective gastruloid clusters. **(C)** Differential gene expression analysis between mouse epiblast from Pijuan-Sala et al. (2019) and corresponding gastruloid clusters (primed pluripotent as well as early differentiation I and II). Genes associated with cell adhesion and migration behaviour are highlighted in red.

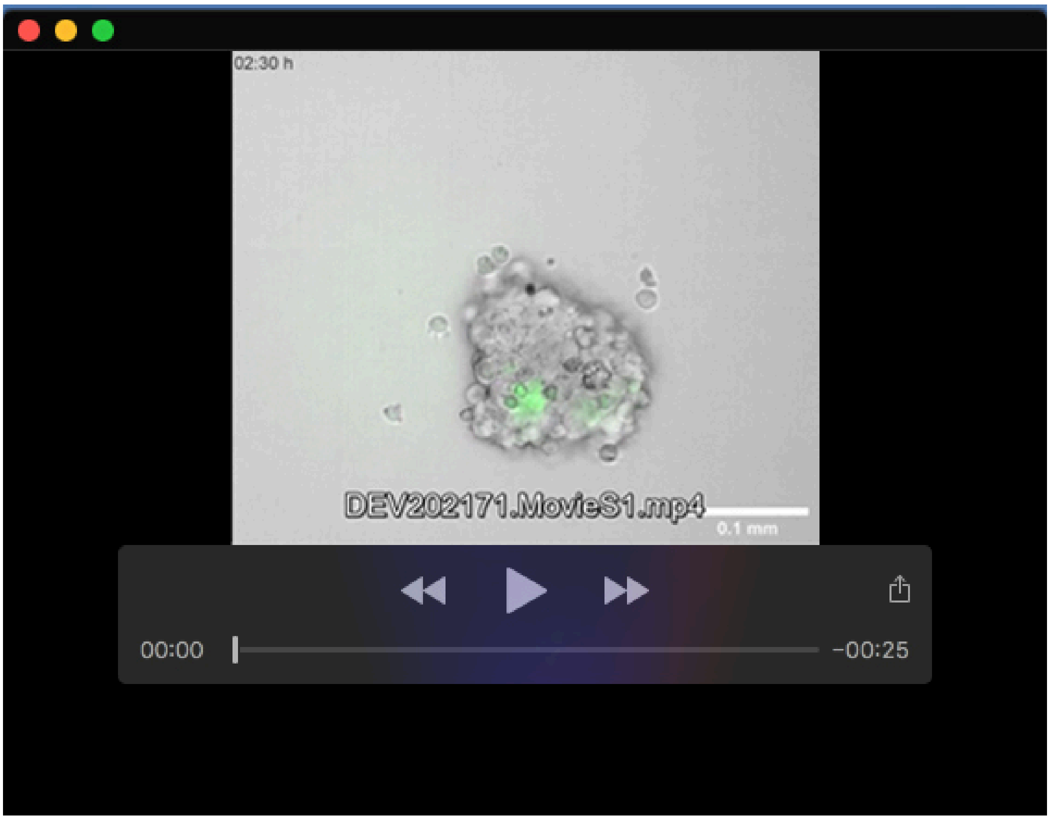

Movie 1. Onset of T expression in gastruloids

Timelapse imaging of T::GFP mESCs aggregated into gastruloids. T channel is displayed in green. n = 15.

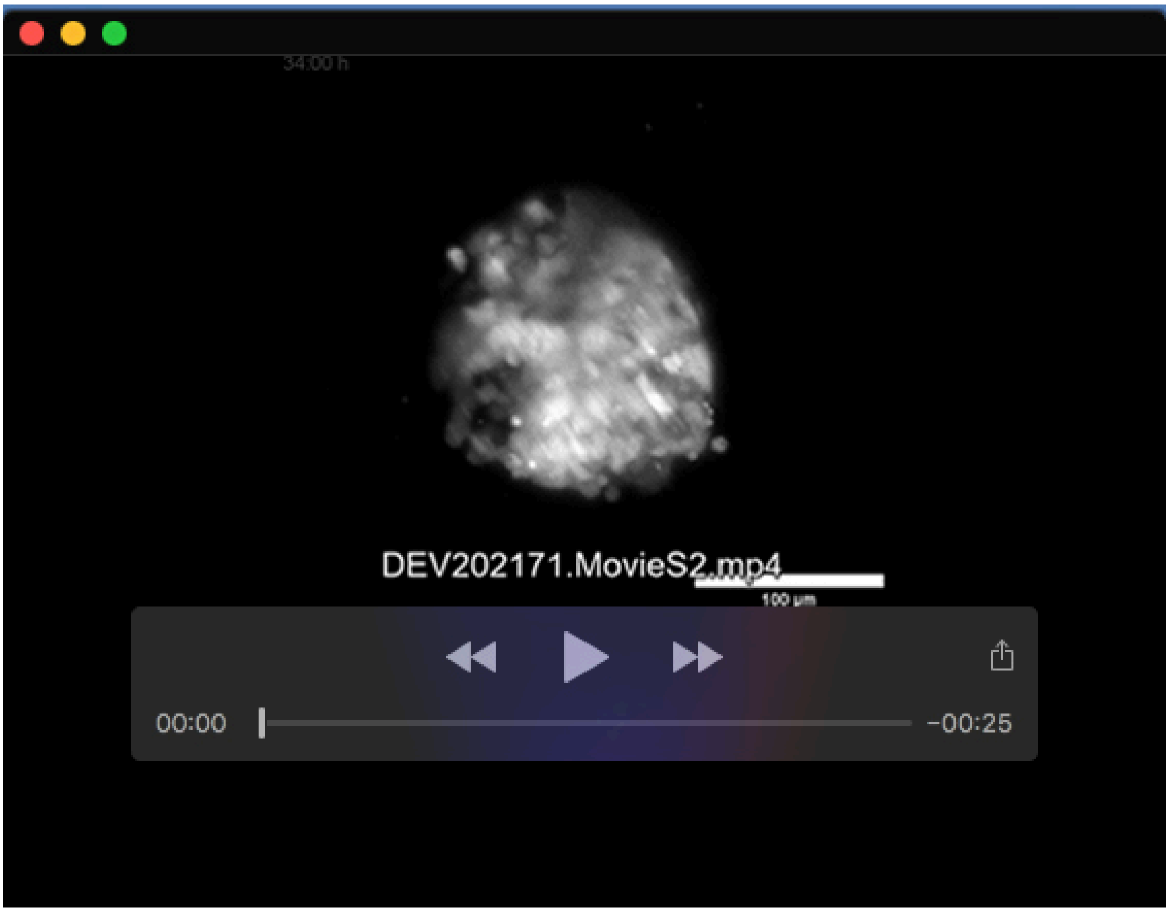

Movie 2. Progression of T polarization

Light sheet live imaging of T::GFP gastruloids (T promoter activity in green) from 24h to 48hpa shows the T symmetry breaking or AP axis formation event. n = 3 with SIR-DNA counterstain and n = 3 without (depicted in this movie).

Development • Supplementary information

Table S1. Quality control parameters.
scRNA-seq quality control parameters for all datasets generated for this study.

Available for download at
https://journals.biologists.com/dev/article-lookup/doi/10.1242/dev.202171#supplementary-data

Table S2. Marker genes genes for clusters in the integrated dataset.
List of the most differentially expressed genes for each cluster in the integrated scRNA-seq dataset (24hpa,48hpa,72hpa), identified via the "FindAllMarkers()" function in Seurat.

Available for download at
https://journals.biologists.com/dev/article-lookup/doi/10.1242/dev.202171#supplementary-data

Table S3. Marker genes for clusters in the 72hpa dataset.
List of the most differentially expressed genes for each cluster in only the 72hpa scRNA-seq dataset, identified via the "FindAllMarkers()" function in Seurat.

Available for download at
https://journals.biologists.com/dev/article-lookup/doi/10.1242/dev.202171#supplementary-data

Table S4. Summary of GO-term analysis results for the 24hpa T^{-} population.
Summary of GO-term analysis results computed via the "ClusterProfiler" "enrichGO()" function.

Available for download at
https://journals.biologists.com/dev/article-lookup/doi/10.1242/dev.202171#supplementary-data

Table S5. Summary of GO-term analysis results for the 24hpa T^{+} population.
Summary of GO-term analysis results computed via the "ClusterProfiler" "enrichGO()" function.

Available for download at
https://journals.biologists.com/dev/article-lookup/doi/10.1242/dev.202171#supplementary-data

Table S6. Summary of GO-term analysis results for the 48hpa T⁻ population. Summary of GO-term analysis results computed via the "ClusterProfiler" "enrichGO()" function.

Available for download at
https://journals.biologists.com/dev/article-lookup/doi/10.1242/dev.202171#supplementary-data

Table S7. Summary of GO-term analysis results for the 48hpa T⁺ population. Summary of GO-term analysis results computed via the "ClusterProfiler" "enrichGO()" function.

Available for download at
https://journals.biologists.com/dev/article-lookup/doi/10.1242/dev.202171#supplementary-data

Table S8. Summary of GO-term analysis results for the 72hpa T⁻ population. Summary of GO-term analysis results computed via the "ClusterProfiler" "enrichGO()" function.

Available for download at
https://journals.biologists.com/dev/article-lookup/doi/10.1242/dev.202171#supplementary-data

Table S9. Summary of GO-term analysis results for the 72hpa T⁺ population. Summary of GO-term analysis results computed via the "ClusterProfiler" "enrichGO()" function.

Available for download at
https://journals.biologists.com/dev/article-lookup/doi/10.1242/dev.202171#supplementary-data

Table S10. Summary of GO-term analysis results for the 24hpa Sox⁻ population. Summary of GO-term analysis results computed via the "ClusterProfiler" "enrichGO()" function.

Available for download at
https://journals.biologists.com/dev/article-lookup/doi/10.1242/dev.202171#supplementary-data

Table S11. Summary of GO-term analysis results for the 24hpa Sox2$^+$ population. Summary of GO-term analysis results computed via the “ClusterProfiler” “enrichGO()” function.

Available for download at
https://journals.biologists.com/dev/article-lookup/doi/10.1242/dev.202171#supplementary-data

Table S12. Summary of GO-term analysis results for the 48hpa Sox2$^-$ population. Summary of GO-term analysis results computed via the “ClusterProfiler” “enrichGO()” function.

Available for download at
https://journals.biologists.com/dev/article-lookup/doi/10.1242/dev.202171#supplementary-data

Table S13. Summary of GO-term analysis results for the 48hpa Sox2$^+$ population. Summary of GO-term analysis results computed via the “ClusterProfiler” “enrichGO()” function.

Available for download at
https://journals.biologists.com/dev/article-lookup/doi/10.1242/dev.202171#supplementary-data

Table S14. Summary of GO-term analysis results for the 72hpa Sox2$^-$ population. Summary of GO-term analysis results computed via the “ClusterProfiler” “enrichGO()” function.

Available for download at
https://journals.biologists.com/dev/article-lookup/doi/10.1242/dev.202171#supplementary-data

Table S15. Summary of GO-term analysis results for the 72hpa Sox2$^+$ population. Summary of GO-term analysis results computed via the “ClusterProfiler” “enrichGO()” function.

Available for download at
https://journals.biologists.com/dev/article-lookup/doi/10.1242/dev.202171#supplementary-data

Supplementary References

Alda-Catalinas, C., Bredikhin, D., Hernando-Herraez, I., Santos, F., Kubinyecz, O., Eckersley-Maslin, M. A., Stegle, O., and Reik, W. (2020). A single-cell transcriptomics crispr-activation screen identifies epigenetic regulators of the zygotic genome activation program. *Cell Systems*, 11(1):25–41.

Nowotschin, S., Setty, M., Kuo, Y.-Y., Liu, V., Garg, V., Sharma, R., Simon, C. S., Saiz, N., Gardner, R., Boutet, S. C., Church, D. M., Hoodless, P. A., Hadjantonakis, A.-K., and Pe'er, D. (2019). The emergent landscape of the mouse gut endoderm at single-cell resolution. *Nature*, 569(7756):361–367.

Pijuan-Sala, B., Griffiths, J. A., Guibentif, C., Hiscock, T. W., Jawaid, W., Calero-Nieto, F. J., Mulas, C., Ibarra-Soria, X., Tyser, R. C. V., Ho, D. L. L., Reik, W., Srinivas, S., Simons, B. D., Nichols, J., Marioni, J. C., and Gottgens, B. (2019). A single-cell molecular map of mouse gastrulation and early organogenesis. *Nature*, 566(7745):490–495.

Development • Supplementary information
