# Supplementary material for: Early autonomous patterning of the anteroposterior axis in gastruloids
Source: Development. 2024 Nov 18;151(22):dev202171. doi: 10.1242/dev.202171 (PMC11607688; doi:10.1242/dev.202171)
Supplement: Supplementary file 1 [file develop-151-202171_review_history.pdf]

Early, autonomous patterning of the anteroposterior axis in gastruloids

Kerim Anlaş, Nicola Gritti, Fumio Nakaki, Laura Salamó Palau, Sham Leilah Tlili, David Oriola, Krisztina Arató, Jia Le Lim, James Sharpe and Vikas Trivedi
DOI: 10.1242/dev.202171

Editor: Matthias Lutolf

Review timeline

Original submission:	11 July 2023
Editorial decision:	11 August 2023
First revision received:	8 February 2024
Editorial decision:	12 March 2024
Second revision received:	4 June 2024
Accepted:	17 June 2024

Original submission

First decision letter

MS ID#: DEVELOP/2023/202171

MS TITLE: Early, autonomous patterning of the anteroposterior axis in gastruloids

AUTHORS: Kerim Anlas, Nicola Gritti, Fumio Nakaki, Laura Salamo Palau, Sham Leilah Tlili, David Oriola, Krisztina Arato, Jia Le Lim, James Sharpe, and Vikas Trivedi

I have now received all the referees' reports on the above manuscript, and have reached a decision. The referees' comments are appended below, or you can access them online: please go to BenchPress and click on the 'Manuscripts with Decisions' queue in the Author Area.

As you will see, the referees express considerable interest in your work, but have some significant criticisms and recommend a substantial revision of your manuscript before we can consider publication. If you are able to revise the manuscript along the lines suggested, which may involve further experiments, I will be happy receive a revised version of the manuscript. Your revised paper will be re-reviewed by one or more of the original referees, and acceptance of your manuscript will depend on your addressing satisfactorily the reviewers' major concerns. Please also note that Development will normally permit only one round of major revision. If it would be helpful, you are welcome to contact us to discuss your revision in greater detail. Please send us a point-by-point response indicating your plans for addressing the referees' comments, and we will look over this and provide further guidance.

Please attend to all of the reviewers' comments and ensure that you clearly highlight all changes made in the revised manuscript. Please avoid using 'Tracked changes' in Word files as these are lost in PDF conversion. I should be grateful if you would also provide a point-by-point response detailing how you have dealt with the points raised by the reviewers in the 'Response to Reviewers' box. If you do not agree with any of their criticisms or suggestions please explain clearly why this is so.

Reviewer 1

© 2024. Published by The Company of Biologists under the terms of the Creative Commons Attribution License (https://creativecommons.org/licenses/by/4.0/).

Advance summary and potential significance to field

That aggregates of embryonic stem cells termed gastruloids can spontaneously break their radial symmetry and arrange cells with different germ layer fates along a coordinate system of body axes has intrigued many stem cell biologists in recent years, but how this symmetry breaking occurs is still not fully understood. In this manuscript, Anlas and colleagues address this question through live imaging protein and mRNA staining, time-course single-cell sequencing and signaling perturbations, focusing particularly on the expression of T/Bra in a reporter cell line as one of the first signs of symmetry breaking. They find surprisingly early heterogeneous T/Bra expression that polarizes within the gastruloid even before a Chiron pulse. Their analysis of cell motility and expression of cell-cell adhesion molecules in T/Bra+-cells leads them to suggest that polarization at the level of gastruloids is a consequence of cell sorting and tissue flows. They also provide new data on lineage specification in gastruloids, show that axial patterning is robust to gastruloid size, and report striking phenotypes upon the timed manipulation of Wnt, FGF and Nodal signaling pathways during gastruloid formation.
Overall, this is a very rich manuscript that reports a set of interesting observation that will be valuable to the gastruloid community, and that meaningfully complements very recent studies addressing similar questions. The data are mostly solid and are presented clearly. Since the work addresses a large range of aspects, including cellular dynamics, signaling control, scaling and lineage differentiation, the analysis of the data sometimes remains somewhat superficial. I highlight specific places and provide recommendations for improvements below. I also feel that the writing could be more concise in several places.

Comments for the author

Major

1. One of the strengths of the present manuscript is that is characterizes early gastruloid development in a different cell line and from different starting conditions compared to other recent studies taking a similar approach (e.g. PMID: 37209681). It thereby shows that protocol details have an impact on the dynamics and trajectories of cell differentiation and gastruloid patterning, and can thus be informative for understanding mechanisms of symmetry breaking. I feel that the manuscript would benefit from a clearer description of different gastruloid protocols in the introduction, and from stating where the current study differs from previous approaches. At the moment this is somewhat scattered throughout the manuscript (e.g. lines 242 – 261).
2. There are several instances where the authors make strong claims, but I am having difficulties finding support for those claims in the figures. For example:
o In line 133 – 135 the authors write “The exact timing of the symmetry breaking may vary between experimental batches”, but they do not provide any data showing this variability.
o In lines 223 – 229, they provide an extensive description of cell behaviors in the text, but these are not obvious from the associated Fig. 1g. I think that differential motility is an attractive explanation for cell sorting and tissue flows, but at the current depth of analysis, this statement is not strongly supported by the data. See also my point 3. below.
o Lines 573, 588/589: The authors mention selected differentially expressed genes, but the corresponding data is difficult to locate in the figures.
3. The authors performed time-resolved light sheet imaging of developing gastruloids, but they only show 2D sections. If they could make use of the 3D nature of their recordings, this could make the paper much stronger, especially when it comes to analyzing the behavior of the T/Bra+ cells in Fig. 1g. This analysis is not absolutely necessary but it could give the manuscript a lift.
4. In the single cell sequencing data, I am missing an explanation how the authors chose a specific resolution for their clustering, and how they determined cluster names for the mesodermal cells. Lines 267 – 277 are somewhat confusing to read; have the different clusters been mixed up?
5. In their scRNAseq data, the authors find a large number of differentiated cells in the starting population (clusters 5, 6, and 7 in Supplementary Fig. 2a b), and observe incomplete differentiation at 48 h and 72 h. I am wondering to which degree this is a general behavior of cells cultured in serum + LIF, and to which degree it reflects the particular behavior of the T/Bra reporter line used in this study. To address this question, the authors could compare the scRNAseq data of their 0 h timepoint to published ESC reference datasets. It could also be informative to

© 2024. Published by The Company of Biologists under the terms of the Creative Commons Attribution License (https://creativecommons.org/licenses/by/4.0/).

assess whether the dynamics of symmetry breaking in gastruloids are the same in the T/Bra line compared to reference cell lines.

6. One of the most intriguing results of the paper is localization of T/Bra+ cells in a banded pattern upon perturbation of Fgf and Tgf-beta signaling. Can the authors give data how often this pattern has been observed, and perhaps speculate how it forms? I am also wondering what is the identity of the Sox2;T/Bra-negative cells (upper right part of the gastruloid shown in Fig. 6f), although I acknowledge that addressing this question might be beyond the scope of this manuscript.

7. In Fig. 7, the authors use a reference dataset spanning from E6.5 to E8.5 for integration of their own scRNAseq data and find that their early time-points do not map well onto that reference. I do not find this surprising, since ESCs cultured in serum + LIF have been shown to be most similar to embryo cells between E4.5 and E5.5 (PMID: 24859004). A more meaningful integration should therefore use an embryo dataset that also contains these earlier stages (e.g. PMID: 30959515), or combine multiple published datasets into a reference that captures all potential stages of the in vitro dataset. Depending on the outcome of this analysis, the authors might need to tone down their conclusion that the same differentiated cell types are reached from different starting conditions (abstract, discussion lines 923 – 929).

Minor

- Fig. 1, legend: Is n=23 the total number of gastruloids, or the (minimum) number per timepoint? Please state this in the figure legend.
- Line 379: There is a reference to a Supp. Fig. 3h, I, but this figure does not exist.
- Movie 2: The nuclear stain in this movie makes the T/Bra reporter signal hard to see. I suspect the very bright signal of the nuclear dye mostly comes from cell debris. The authors may want to consider showing a gastruloid without nuclear stain, or only use the nuclear stain to indicate the outlines of the gastruloid.
- Figure 6: Please state the number of independent experiments, and number of gastruloids per experiment. For panel c, consider showing control gastruloids.
- Ref [62] has now been published in a journal with PMID: 35707767
- Gastruloids at 24 h (and sometimes also at 48 h) appear to be round. How is an AP axis defined under these conditions, and how can it be oriented? Is it possible that by aligning random variations, the analysis creates the (wrong) impression of consistent symmetry breaking?
- Line 1304: A word seems to be missing here.
- Supp Fig. 1e: This figure has Insufficient resolution to see the rosettes mentioned in the text.

Reviewer 2

Advance summary and potential significance to field

Anlaş, Gritti and colleagues provide here a well-performed study on gastruloids, a topical in vitro model of embryonic development. They investigate the dynamic of gastruloid anteroposterior axis development and its robustness to a variety of experimental perturbations.
The experiments conducted and the resulting data appear to be of commendable quality, potentially substantiating their assertions. The variety of experiments presented here are interesting and help us better understand the ability of gastruloid to form their antero-posterior axis. The major advantage of this manuscript are its experiments which seem well designed and of good quality.

Comments for the author

However, reading this manuscript was challenging, and connecting the text with the figure was even more so. A more coherent sequence of ideas would facilitate the reader's comprehension and strengthen the central message. This could involve streamlining the content and accentuating a clear, central research question, which would undoubtedly elevate the manuscript's readability and impact.
Again, I acknowledge the evident efforts that went in this work; but the manuscript would benefit from increased focus and/or better organization.

© 2024. Published by The Company of Biologists under the terms of the Creative Commons Attribution License (https://creativecommons.org/licenses/by/4.0/).

In light of this, I would recommend a deep reconsideration of the manuscript organization to ensure a more coherent flow of ideas. Streamlining the content and emphasizing a clear and central research question would undoubtedly enhance the manuscript's impact and readability.
At this point, despite the commendable quality and array of data presented here, it is difficult for me to envision a pathway to publication without major revisions to the manuscript's structure. The current format has hindered my ability to thoroughly assess and evaluate the findings.
Major comments:
Throughout the manuscript, there is an omnipresent lack of clarity that stems from different issues in the way the manuscript is written. Notably, the absence of adequate figure citations at times, hinders the ability to correlate specific data with corresponding claims In addition, the order of the sections in the result portion do not help with the clarity either. The transition between sections is not obvious. For example the authors go from their well-performed single cell RNA-seq time course to their precise developmental pathway perturbation analyses back to their single cell analysis. However, initially the authors make an important point on the different pluripotent populations in gastruloids (Fig 2 and Supp Fig 2) and wait the end of the manuscript to follow this observation directly and compare it to the mouse embryo, Fig. 7, Supp Fig 9-10). It would likely help conveying this message to order these sections one after the other.
Finally, the single cell comparison with the mouse embryo shows discrepancies between two methods of aligning multiple datasets (Seurat integrate vs MNN). The Seurat integrate approach shown in Supp Fig 9 shows good correlation between the G primed pluripotent and Epiblast (r=0.98) and G Early Diff correlating with the Primitive streak (0.97). However indeed the MNN integration fail to associated these identities. It is difficult to understand why the authors here chooses to put more trust in the method stating one result vs the other. If anything, this discrepancy should prevent you from drawing these types of conclusions unless alternative methodologies are used.
I will provide here a few examples:
L175-177: Contrary to what is written, at 48h a structure with high phalloidin staining seems clearly apparent (Supp Fig 1d), are such structure reproducibly present? Can the author comment on what this structure may be?
L212-214: could the author provide the quantification of the motility of T+ for this statement or refer to a figure showing this (or rephrase this statement)?
L232-235: The connection between this statement and the presented data isn't clear to me.
L278-280: the authors make a strong (and likely correct) statement on their observation that late stage gastruloid maintain a naive pluripotent population in contrast with the primed pluripotent, could they show the expression of genes that are associated with naive pluripotency (e.g. Zfp42 which is present but annotated as pluripotent instead of naive vs primed pluripotency) and genes associated with primed pluripotency such as the ones they cite in line: 269-
272). in the figures they only write pluripotent instead of differentiating the markers between primed vs naïve.
L290-291: This is likely true but citing a figure showing it would help.
L292-296: Could the author cite the figure corresponding to this data? I suppose the author meant Section 3.8.1. Either way, I am not sure which data is used to drive the conclusion that “the rate of differentiation from primed to T+
determines the timescale of symmetry breaking”. But either way, for readability it is odd to have to go to the text placed later to understand what is meant here and it might be better to keep this observation for later.
L321-333: Here, most of the genes mentioned here are not present in the figure only Epha5 and Sox17 are shown. Instead, Zf42, Sox2, Sox1, Bra are shown but not mentioned in the text.
L365-366: it is not clear what the authors mean by autonomous here.
L529-532: In the context of the simultaneous timing with T, it would be more appropriate to cite Fig. 5d for this which supports this much more clearly than Fig sup 5a L577-585: This observation could help introduce section 3.8.1, so as a suggestion, this could be placed instead at the end of the paragraph right before section 3.8.1 L627-6280: it's not clear to me which data shows the reduction of T reporter level and its recovery by 72hpa when treatment with XAV939 is done between 0 and 24hpa (The kymograph in Fig6b only show 24-48hpa and not 0-24hpa). The recovery of T reporter intensity is clear at 72h however, but localization seems much more homogeneous but this is not mentioned.
Minor comments:
L178-179: The rosettes aren't really apparent in the provided picture, could the author comment on their frequency and provide a representative picture?

© 2024. Published by The Company of Biologists under the terms of the Creative Commons Attribution License (https://creativecommons.org/licenses/by/4.0/).

L285-288: To complement this statement, showing as a supplemental figure a picture of the embryonic stem cells cultivated in SL condition by the authors could be useful to evaluate the differentiation status of the cells analysed here.
L352: do the author have a citation or data which can be used to make this connection with the e6.5 embryo?
L513-516: Unless the authors have some data supporting this claim, an alternative explanation could also be that cell lineages can emerge in a random probabilistic manner followed by a cell-sorting mechanism.
L554-556: the authors mention the expression of genes involved in EMT, showing the expression of Snai1, Cdh1 and Cdh2 should be shown here (in T+ vs T-to document this)
L754: In Supp Fig 9a, very few cells appear to come from the G Pluripotent I population while they were quite numerous in Fig2a, and these cells which did not map onto the embryo UMAP?

First revision

Author response to reviewers' comments

We thank the reviewers for the feedback and will answer the individual points in detail below. We have also marked the major changes in red in the revised manuscript for the ease of following changes.

Reviewer 1

Advance Summary and Potential Significance to Field:
That aggregates of embryonic stem cells termed gastruloids can spontaneously break their radial symmetry and arrange cells with different germ layer fates along a coordinate system of body axes has intrigued many stem cell biologists in recent years, but how this symmetry breaking occurs is still not fully understood. In this manuscript, Anlas and colleagues address this question through live imaging, protein and mRNA staining, time-course single-cell sequencing and signaling perturbations, focusing particularly on the expression of T/Bra in a reporter cell line as one of the first signs of symmetry breaking. They find surprisingly early heterogeneous T/Bra expression that polarizes within the gastruloid even before a Chiron pulse. Their analysis of cell motility and expression of cell-cell adhesion molecules in T/Bra+-cells leads them to suggest that polarization at the level of gastruloids is a consequence of cell sorting and tissue flows. They also provide new data on lineage specification in gastruloids, show that axial patterning is robust to gastruloid size, and report striking phenotypes upon the timed manipulation of Wnt, FGF and Nodal signaling pathways during gastruloid formation.
Overall, this is a very rich manuscript that reports a set of interesting observation that will be valuable to the gastruloid community, and that meaningfully complements very recent studies addressing similar questions. The data are mostly solid and are presented clearly. Since the work addresses a large range of aspects, including cellular dynamics, signaling control, scaling and lineage differentiation, the analysis of the data sometimes remains somewhat superficial. I highlight specific places and provide recommendations for improvements below. I also feel that the writing could be more concise in several places.

Reviewer 1 Comments for the Author:
Major

1. One of the strengths of the present manuscript is that is characterizes early gastruloid development in a different cell line and from different starting conditions compared to other recent studies taking a similar approach (e.g. PMID: 37209681). It thereby shows that protocol details have an impact on the dynamics and trajectories of cell differentiation and gastruloid patterning, and can thus be informative for understanding mechanisms of symmetry breaking. I feel that the manuscript would benefit from a clearer description of different gastruloid

© 2024. Published by The Company of Biologists under the terms of the Creative Commons Attribution License (https://creativecommons.org/licenses/by/4.0/).

protocols in the introduction, and from stating where the current study differs from previous approaches. At the moment this is somewhat scattered throughout the manuscript (e.g. lines 242 – 261).

We thank the reviewer for the feedback and we have clarified these aspects in the revised version of the manuscript.

2. There are several instances where the authors make strong claims, but I am having difficulties finding support for those claims in the figures. For example: oIn line 133 – 135 the authors write “The exact timing of the symmetry breaking may vary between experimental batches”, but they do not provide any data showing this variability.

We agree that this sentence could be confusing and we have now corrected that.

oIn lines 223 – 229, they provide an extensive description of cell behaviours in the text, but these are not obvious from the associated Fig. 1g. I think that differential motility is an attractive explanation for cell sorting and tissue flows, but at the current depth of analysis, this statement is not strongly supported by the data. See also my point 3. Below.

Looking at the uploaded manuscript files we have now realized that many of the questions raised by the two reviewers, including this one, originate from the fact that the images were compressed making the data less self-evident. Having said this, we agree with the reviewer and can now revise the panel as follows to address the concerns:

(a) We have added distinct arrows to point to areas of particular interest.
(b) We have quantified the displacement of several T+ cells which is to be included in the respective Supplementary Figure.
(c) Furthermore, we can rephrase the text in a more cautious manner regarding our conclusions on cell-sorting events.

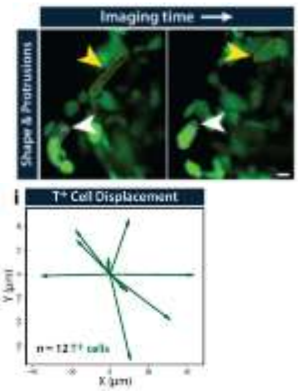

oLines 573, 588/589: The authors mention selected differentially expressed genes, but the corresponding data is difficult to locate in the figures.

This was a mistake and the text has to be linked to figure 5a,b

3. The authors performed time-resolved light sheet imaging of developing gastruloids, but they only show 2D sections. If they could make use of the 3D nature of their recordings, this could make the paper much stronger, especially when it comes to analyzing the behavior of the T/Bra+ cells in Fig. 1g. This analysis is not absolutely necessary but it could give the manuscript a lift.

We intentionally chose to showcase 2D cross-sections because a dense collection of cytoplasmic cell labels is difficult to visualize in 3D. Since we wanted to showcase the richness of cell behaviour (movement, shape changes, protrusions, divisions) that are concomitant with symmetry-breaking and showing 2D cross-sections of the light-sheet imaging (SPIM) was thus necessary for that. We are confident that the additional figure panels (as mentioned above) and improved resolution will convey these aspects much better than what we have in the current version.

© 2024. Published by The Company of Biologists under the terms of the Creative Commons Attribution License (https://creativecommons.org/licenses/by/4.0/).

4. In the single cell sequencing data, I am missing an explanation how the authors chose a specific resolution for their clustering, and how they determined cluster names for the mesodermal cells. Lines 267 – 277 are somewhat confusing to read; have the different clusters been mixed up?

We have addressed these aspects in the revised version. In a nutshell the text is now simplified to explain the choice of clusters. We direct the reader to a new supplementary figure (see a subset of those panels below) and to the existing Fig 2e where we show that the designated clusters show sufficiently distinct gene expressions. It must be noted that despite our efforts to use an objective criterion for deciding the number of clusters, the choice in the end is of qualitative nature as the number of visually distinct clusters is very dataset (sample types) dependent and it is, therefore, a general practice for researchers to make the final decision depending upon the biological question, something that we did as well.

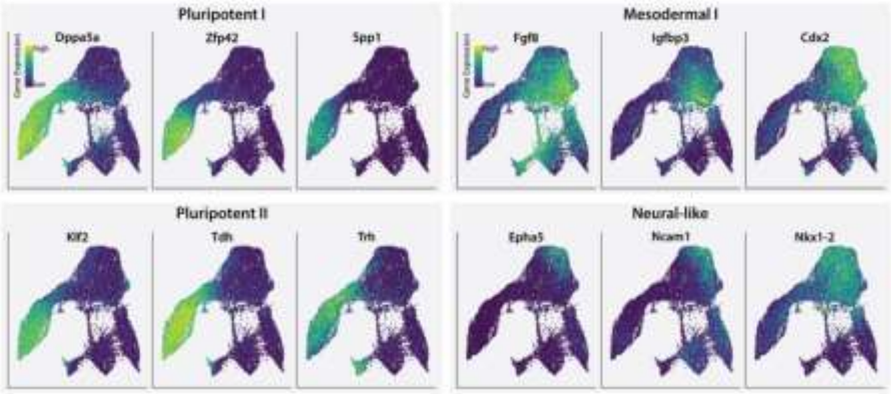

5. In their scRNAseq data, the authors find a large number of differentiated cells in the starting population (clusters 5, 6, and 7 in Supplementary Fig. 2a,b), and observe incomplete differentiation at 48 h and 72 h. I am wondering to which degree this is a general behavior of cells cultured in serum + LIF, and to which degree it reflects the particular behavior of the T/Bra reporter line used in this study. To address this question, the authors could compare the scRNAseq data of their 0 h timepoint to published ESC reference datasets. It could also be informative to assess whether the dynamics of symmetry breaking in gastruloids are the same in the T/Bra line compared to reference cell lines.

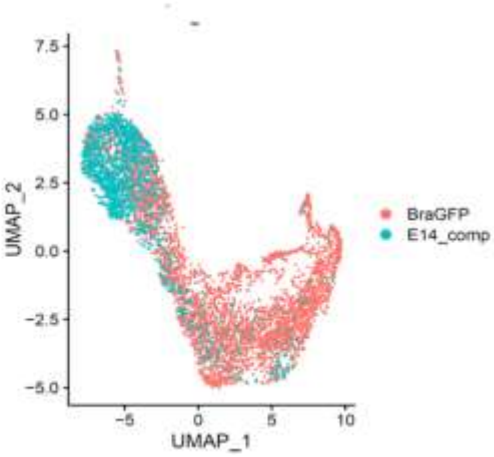

We thank the reviewer for this point as it is a very valid issue. To address this we have now also used the dataset from https://pubmed.ncbi.nlm.nih.gov/32634384/ and https://www.ncbi.nlm.nih.gov/geo/query/acc.cgi?acc=GSM4017119 where the cell line used was 129/Ola ES-E14 WT mESC line, also maintained in DMEM-based serum/LIF culture media. Integrating this dataset with our "0h" mESCs (Bra::GFP reporter, E14 background), we find that the cells map on top of each other. Our Bra::GFP cells, though, comprise of a higher fraction of cells in primed pluripotent to early

© 2024. Published by The Company of Biologists under the terms of the Creative Commons Attribution License (https://creativecommons.org/licenses/by/4.0/).

differentiated states/clusters. This suggests that, while the two cell lines are in principle comparable, some mESC lines may exhibit more heterogeneous pluripotency levels even when maintained under similar conditions. Notably, results obtained with the Bra::GFP cell line (and other lines by other labs) appear to be internally consistent across experimental batches, as seen, for example, in our case (see quantifications, numerous HCR staining and especially Supplementary Figure 2c and d.) This may further help to understand differences in observations in gastruloids from different labs, given the composition of source mESCs with respect to primed subpopulations, despite similar culture conditions.

6. One of the most intriguing results of the paper is localization of T/Bra+cells in a banded pattern upon perturbation of Fgf and Tgf-beta signaling. Can the authors give data how often this pattern has been observed, and perhaps speculate how it forms? I am also wondering what is the identity of the Sox2;T/Bra-negative cells (upper right part of the gastruloid shown in Fig. 6f), although I acknowledge that addressing this question might be beyond the scope of this Manuscript.

Again, this is much-appreciated feedback but as the reviewer pointed out this is beyond the scope of this manuscript. Gaining a mechanistic understanding will require many more experiments that we are pursuing in the lab and therefore we have proposed to now remove this section. This is also because we had already exceeded the prescribed word limits in our original submission (1882 words above the limit for a Research Article) and now with the changes suggested by reviewers, we were going even above the current word count. Therefore, we felt that the best way forward (without having to split the manuscript, should it be accepted) was to remove the results on signaling perturbations and its corresponding figure (Figure 6, originally)). This will allow us to save these observations for a follow-up manuscript where we do an in-depth analysis of the phenotypes under the perturbation regimes.

7. In Fig. 7, the authors use a reference dataset spanning from E6.5 to E8.5 for integration of their own scRNAseq data and find that their early time-points do not map well onto that reference. I do not find this surprising, since ESCs cultured in serum + LIF have been shown to be most similar to embryo cells between E4.5 and E5.5 (PMID: 24859004). A more meaningful integration should therefore use an embryo dataset that also contains these earlier stages (e.g. PMID: 30959515), or combine multiple published datasets into a reference that captures all potential stages of the in vitro dataset. Depending on the outcome of this analysis, the authors might need to tone down their conclusion that the same differentiated cell types are reached from different starting conditions (abstract, discussion lines 923 – 929).

We appreciate the reviewer raising this crucial point and have leveraged the dataset they suggested in order to perform an (MNN- based) integration with the same parameters as in our manuscript.

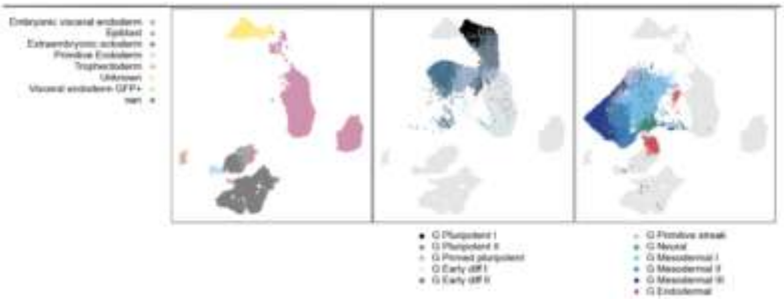

Since the mouse data is for E4.5-E5.5 stages the integration clearly shows that

(a) there is some degree of mapping particularly of the most pluripotent gastruloid cell

© 2024. Published by The Company of Biologists under the terms of the Creative Commons Attribution License (https://creativecommons.org/licenses/by/4.0/).

populations to the putative mouse ICM (in the epiblast cluster) and the extra-embryonic clusters remain unmapped. Note that, unfortunately, the mouse reference dataset is only partially annotated (see below the “unknown” cluster which we assume to be ICM).

(b) more primed and early differentiated gastruloid cells, once again, map outside of the mouse data.

Overall, this corroborates our findings in the manuscript and further specifies that gastruloid (naive) pluripotent cells resemble the ICM somewhat more closely, however the primed and early differentiating cells nevertheless differ (transcriptionally).

Minor
•Fig. 1, legend: Is n=23 the total number of gastruloids, or the (minimum) number per timepoint? Please state this in the figure legend.
•Line 379: There is a reference to a Supp. Fig. 3h, I, but this figure does not exist.
•Movie 2: The nuclear stain in this movie makes the T/Bra reporter signal hard to see. I suspect the very bright signal of the nuclear dye mostly comes from cell debris. The authors may want to consider showing a gastruloid without nuclear stain, or only use the nuclear stain to indicate the outlines of the gastruloid.
•Figure 6: Please state the number of independent experiments, and number of gastruloids per experiment. For panel c, consider showing control gastruloids.
•Ref [62] has now been published in a journal with PMID: 35707767
•Gastruloids at 24 h (and sometimes also at 48 h) appear to be round. How is an AP axis defined under these conditions, and how can it be oriented? Is it possible that by aligning random variations, the analysis creates the (wrong) impression of consistent symmetry breaking?
•Line 1304: A word seems to be missing here. •Supp Fig. 1e: This figure has Insufficient resolution to see the rosettes mentioned in the text.

All of these minor points have been addressed in the revised manuscript.

Reviewer 2

Advance Summary and Potential Significance to Field:
Anlaș, Gritti and colleagues provide here a well-performed study on gastruloids, atopical in vitro model of embryonic development. They investigate the dynamic of gastruloid anteroposterior axis development and its robustness to a variety of experimental perturbations.
The experiments conducted and the resulting data appear to be of commendable quality, potentially substantiating their assertions. The variety of experiments presented here are interesting and help us better understand the ability of gastruloid to form their antero-posterior axis. The major advantage of this manuscript are its experiments which seem well designed and of good quality.

Reviewer 2 Comments for the Author:
However, reading this manuscript was challenging, and connecting the text with the figure was even more so. A more coherent sequence of ideas would facilitate the reader's comprehension and strengthen the central message. This could involve streamlining the content and accentuating a clear, central research question, which would undoubtedly elevate the manuscript's readability and Impact. Again, I acknowledge the evident efforts that went in this work; but the manuscript would benefit from increased focus and/or better organization.
In light of this, I would recommend a deep reconsideration of the manuscript organization to ensure a more coherent flow of ideas. Streamlining the content and emphasizing a clear and central research question would undoubtedly enhance the manuscript's impact and readability.
At this point, despite the commendable quality and array of data presented here, it is difficult for me to envision a pathway to publication without major revisions to the manuscript's structure. The current format has hindered my ability to thoroughly assess and evaluate the findings.

Major comments:

© 2024. Published by The Company of Biologists under the terms of the Creative Commons Attribution License (https://creativecommons.org/licenses/by/4.0/).

Throughout the manuscript, there is an omnipresent lack of clarity that stems from different issues in the way the manuscript is written. Notably, the absence of adequate figure citations at times, hinders the ability to correlate specific data with corresponding claims
In addition, the order of the sections in the result portion do not help with the clarity either. The transition between sections is not obvious. For example, the authors go from their well-performed single cell RNA-seq time course to their precise developmental pathway perturbation analyses back to their single cell analysis. However, initially the authors make an important point on the different pluripotent populations in gastruloids (Fig 2 and Supp Fig 2) and wait the end of the manuscript to follow this observation directly and compare it to the mouse embryo, Fig. 7, Supp Fig 9-10). It would likely help conveying this message to order these sections one after the other.

We agree about the inadequacy of figure citations. In the revised version, we have addressed this.

We thank the reviewer for appreciating our scRNA-seq time course and the precise developmental pathway perturbations. However, the suggestion to rearrange the sections is a major one and since Reviewer 1 did not raise this concern we would like to propose the following two options before making changes. Please note that (as explained in response to point 6 of reviewer 1) the figure on signalling perturbations have now been removed, and this helps with the flow of the text as well.

Our current rationale for the flow of information was as follows (we will refer to it as ***Option A***):

- *Figure 1*: We first described the dynamics and global pattern of symmetry-breaking at tissue level
- *Figure 2*: We then investigated the emergence of different cell types through scRNA-seq time course
- *Figures 3 and 4*: From the sCRNASeq dataset, we then probe deeper to look into markers of AP axial patterning in gastruloids prior to external Wnt stimulation, perform HCR stains to see their spatial patterns and then test if such a spatial arrangement of gene expression domains dependent on size.
- *Figure 5*: The robustness of symmetry breaking thus observed is then related to distinct transcriptional cell states between T^+ and T^- populations.
- *Figure 6*: Finally, we use our scRNASeq data to compare the cell types emerging in the gastruloids to those in the developing embryo. We find that cell types that emerge in gastruloids largely map on to those in the embryo. That is encouraging and justifies the use of this system to probe mechanisms of symmetry breaking through relative spatial patterns of gene expression domains, signaling and size dependence (*Figures 3-6*). However, we also find that mESC start from different state as compared to the pluripotent cells in the embryo which is what we demonstrate and this opens up the *discussion section* that concludes the paper.

If the above-mentioned flow of information is not easily accessible, we can re-arrange the paper figures to provide information in the following way (we will refer to it as ***Option B***):.

- *Figure 1*: We first describe the dynamics and global pattern of symmetry-breaking at tissue level (Currently also Figure 1)
- *Figure 2*: Since the symmetry-breaking is defined as emergence of a T^+ pole, we look into the distinct transcriptional cell states between T^+ and T^- populations (This is currently Figure 5 but moving these figures earlier in the paper following by our results on scRNASeq data should address the concern that "*authors go from their well-performed single cell RNA-seq time course to their precise developmental pathway perturbation analyses back to their single cell analysis*).
- *Figure 3*: Our results on scRNA-seq time course (Currently Figure 2)

© 2024. Published by The Company of Biologists under the terms of the Creative Commons Attribution License (https://creativecommons.org/licenses/by/4.0/).

- *Figures 4 and 5*: We probe markers of AP axial patterning in gastruloids, perform HCR stains and size-dependent (Currently Figures 3 and 4).
- *Figure 6*: Comparison with the embryo (Currently also Figure 7) followed by the *discussion section* that concludes the paper.

Finally, the single cell comparison with the mouse embryo shows discrepancies between two methods of aligning multiple datasets (Seurat integrate vs MNN). The Seurat integrate approach shown in Supp Fig 9 shows good correlation between the G primed pluripotent and Epiblast (r=0.98) and G Early Diff correlating with the Primitive streak (0.97). However indeed the MNN integration fail to associated these identities. It is difficult to understand why the authors here chooses to put more trust in the method stating one result vs the other. If anything, this discrepancy should prevent you from drawing these types of conclusions unless alternative methodologies are used.

This is an important aspect and although we tried to address these in our text (Lines 1500-1507) the reviewer's question make it clear that the writing and particularly the figure legend needs to be improved. The label transfer workflow entails going into PC-space, doing batch correction on the datasets, MNN analysis and then the actual label transfer. Accordingly, the correlation coefficients are based on PC- space, but computed before the (final step) label transfer. Hence the good correlations only demonstrate that G primed pluripotent and M epiblast are the most similar populations among the "available" ones, but do not necessarily indicate that it is indeed appropriate to only perform label transfer and not to opt for the more unbiased MNN-integration: The additional measures we compute to assess the label transfer step in Supp Fig 9 e-g (and explained in lines 1513-1532) argue that some G cell populations tend to map to relatively few unique M cells and may also exhibit lower "labelling agreement" or "score". Furthermore, to us, the core observation is not so much that G primed and early differentiating cells map not so well onto the M epiblast and primitive streak in the integrated (MNN-based) UMAP of Main Figure 7a but rather that they seem to exhibit a more mesenchymal transcriptome (as described in lines 795-830) which probably underlies this poor mapping. The observation itself that they do not map properly (using the MNN-only approach) thereby prompts this key, subsequent direct transcriptional comparison which is independent and will yield the same results regardless of the integration method chosen.

I will provide here a few examples:L175-177: Contrary to what is written, at 48h a structure with high phalloidin staining seems clearly apparent (Supp Fig 1d), are such structure reproducibly present? Can the author comment on what this structure may be?

L232-235: The connection between this statement and the presented data isn't clear to me.

These are all valid concerns and we have addressed these in the revised version.
L212-214: could the author provide the quantification of the motility of T+ for this statement or refer to a figure showing this (or rephrase this statement)?

We are providing the following figure in support.

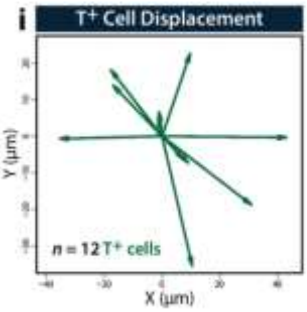

© 2024. Published by The Company of Biologists under the terms of the Creative Commons Attribution License (https://creativecommons.org/licenses/by/4.0/).

L278-280: the authors make a strong (and likely correct) statement on their observation that late stage gastruloid maintain a naive pluripotent population, in contrast with the primed pluripotent, could they show the expression of genes that are associated with naive pluripotency (e.g. Zfp42 which is present but annotated as pluripotent instead of naive vs primed pluripotency) and genes associated with primed pluripotency such as the ones they cite in line: 269-272). in the figures they only write pluripotent instead of differentiating the markers between primed vs naïve.

While we had displayed one naïve and one pluripotent marker in Fig 2b, we have added an additional panel in the Supp info to make this point clear as follows

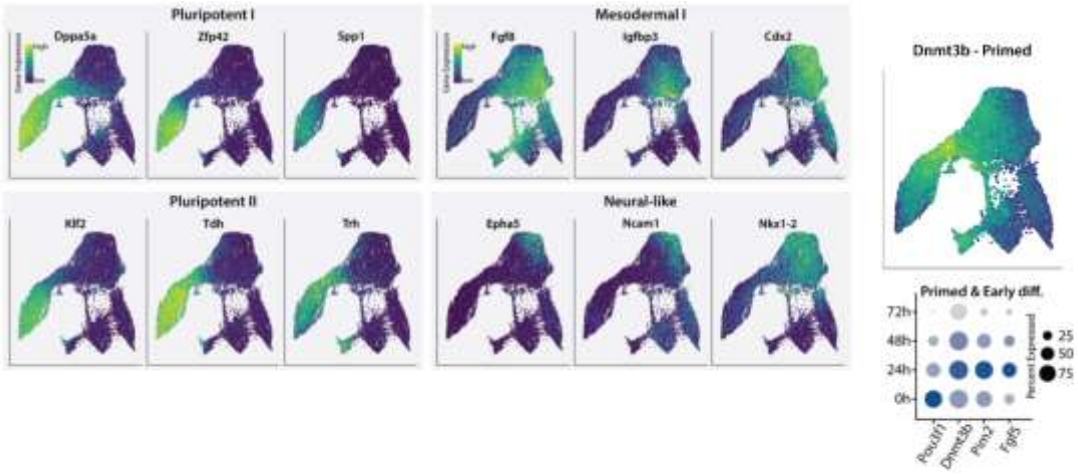

L290-291: This is likely true but citing a figure showing it would help.

We will cite Supp Fig 1a in the main text to address this.

L292-296: Could the author cite the figure corresponding to this data? I suppose the author meant Section 3.8.1. Either way, I am not sure which data is used to drive the conclusion that "the rate of differentiation from primed to T+ determines the timescale of symmetry breaking". But either way, for readability, it is odd to have to go to the text placed later to understand what is meant here and it might be better to keep this observation for later.

This is a valid point but now with the removal of figure on signalling perturbation this has been addressed.

L321-333: Here, most of the genes mentioned here are not present in the figure, only Epha5 and Sox17 are shown. Instead, Zf42, Sox2, Sox1, Bra are shown but not mentioned in the text.

There is indeed a new Supp. Fig with key cluster marker genes (including the genes mentioned by the reviewer) shown on the UMAP as explained before. The genes have also been adapted to increase clarity.

L365-366: it is not clear what the authors mean by autonomous here.

We meant without Chiron/any endoderm inducing pulse. We state this explicitly.

L529-532: In the context of the simultaneous timing with T, it would be more appropriate to cite Fig. 5d for this which supports this much more clearly than Fig sup 5a

Yes, we have cited both.

L577-585: This observation could help introduce section 3.8.1, so as a suggestion, this could be placed instead at the end of the paragraph right before section 3.8.1

Yes, we have incorporated the suggested change.

L627-6280: it's not clear to me which data shows the reduction of T reporter level and its recovery

© 2024. Published by The Company of Biologists under the terms of the Creative Commons Attribution License (https://creativecommons.org/licenses/by/4.0/).

by 72hpa when treatment with XAV939 is done between 0 and 24hpa (The kymograph in Fig6b only show 24-48hpa and not 0-24hpa). The recovery of T reporter intensity is clear at 72h however, but localization seems much more homogeneous but this is not mentioned.

We have adapted the text for clarity and have added a panel to the respective Supplementary Figure to corroborate this point.

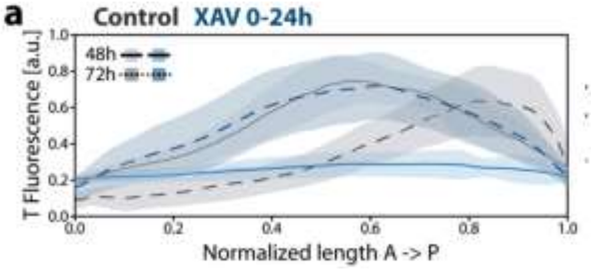

Minor comments:
L178-179: The rosettes aren't really apparent in the provided picture, could the author comment on their frequency and provide a representative picture?

L285-288: To complement this statement, showing as a supplemental figure a picture of the embryonic stem cells cultivated in SL condition by the authors could be useful to evaluate the differentiation status of the cells analysed

L352: do the author have a citation or data which can be used to make this connection with the e6.5 embryo?
L513-516: Unless the authors have some data supporting this claim, an alternative explanation could also be that cell lineages can emerge in a random probabilistic manner followed by a cell-sorting mechanism.

L554-556: the authors mention the expression of genes involved in EMT, showing the expression of Snai1, Cdh1 and Cdh2 should be shown here (in T+ vs T-to document this)

L754: In Supp Fig 9a, very few cells appear to come from the G Pluripotent I population while they were quite numerous in Fig2a, and these cells which did not map onto the embryo UMAP?

These are all questions/suggestions that have been addressed in the revised manuscript.

Second decision letter

MS ID#: DEVELOP/2023/202171

MS TITLE: Early, autonomous patterning of the anteroposterior axis in gastruloids

AUTHORS: Kerim Anlas, Nicola Gritti, Fumio Nakaki, Laura Salamo Palau, Sham Leilah Tlili, David Oriola, Krisztina Arato, Jia Le Lim, James Sharpe, and Vikas Trivedi

I have now received all the referees reports on the above manuscript, and have reached a decision. The referees' comments are appended below, or you can access them online: please go to BenchPress and click on the 'Manuscripts with Decisions' queue in the Author Area.

The overall evaluation is positive and we would like to publish a revised manuscript in Development, provided that the referees' comments can be satisfactorily addressed. Please attend

© 2024. Published by The Company of Biologists under the terms of the Creative Commons Attribution License (https://creativecommons.org/licenses/by/4.0/).

to all of the reviewers' comments in your revised manuscript and detail them in your point-by-point response. If you do not agree with any of their criticisms or suggestions explain clearly why this is so. If it would be helpful, you are welcome to contact us to discuss your revision in greater detail. Please send us a point-by-point response indicating your plans for addressing the referees' comments, and we will look over this and provide further guidance.

Reviewer 1

Advance summary and potential significance to field

For a summary of the contents of the paper see my review from the initial submission. The main change compared to the original submission is the removal of one main figure and the corresponding text describing the effects of timed signaling perturbations on gastruloid development. While these experiments gave very intriguing phenotypes, the authors were unable to come up with a mechanistic explanation, and to meaningfully link these observations with the other data in the original version of the manuscript. In the interest of flow and readability, I agree with the author's decision to remove this section.
In addition, the authors have made a number of smaller changes, mostly through revisions to the text, but also through addition of new supplementary figures where they quantify the motility of T/Bra+ cells, compare gene expression in pluripotency between their own line and a published dataset, and showcase expression patterns of marker genes in UMAP space.
With the changes made in the revision, the manuscript is now much more readable and focused on an important question – the emergence of cellular diversity during early gastruloid development. By highlighting the importance of initial culture conditions, it nicely complements other recent studies on the same topic, and is therefore suitable for publication in Development.

Comments for the author

Most of my points have been appropriately addressed. In a final version, the authors may want to consider two minor additions:

1. The quantification of the motility of T/Bra+ cells in the new Supp. Fig. 1i is nice. Can the authors test whether the motility of T/Bra-negative cells is different?

2. I am still missing an explanation (in the methods section) how an axis was determined in gastruloids at 24h and 48h that are still mostly round (minor point 6. from the initial round of reviews).

Reviewer 2

Advance summary and potential significance to field

Anlaş, Gritti and colleagues provided here a well-performed study on gastruloids a topical in vitro model of embryonic development where they show that lineage specification and patterning can occur prior (and in absence) of external cues (CHIR). The authors have done a commendable job in improving their manuscript and its readability. I am now in support of going forwards with a publication.

Comments for the author

The authors make a correct assessment that rearranging the text in a major fashion would constitute a major revision, and I think removing the signaling perturbations made the manuscript much more fluid so the option A which is the current one sufficiently conveys their message.
I think the authors have done a tremendous job improving their manuscript and its readability, the comparison with the early mouse single cell dataset is compelling although, it is surprising to see so little overlap since other datasets seem to show better correspondence with mouse epiblast (Suppinger et al 2023), maybe this is due to the ESC culture conditions.

© 2024. Published by The Company of Biologists under the terms of the Creative Commons Attribution License (https://creativecommons.org/licenses/by/4.0/).

The point made by the authors regarding media composition from seemingly similar denotation (ESL) while using different actual media GMEM, DMEM +KO or KO DMEM is an important one, and may very well explain the different behavior observed between the line used in this study and the E14tg2a shown in Fig. supp. 2.

minor comments:
Supp. Fig. 1e isn't cited l. 151 and Figure 1c: the authors state a limited overlap between T and Sox2 signal, however it seems that the Sox2 signal (in the quantification) is quite homogeneous at that stage while T already shows the initiation of polarization.
In contrast, as stated l. 152-155, the 48hpa quantification is clearly showing spatial segregation of the two. Maybe one could phrase line 151 as: at 24hpa while T signal already show polarization, Sox2 signal appears more homogeneous throughout gastruloids.
l. 164 typo where two "." are present.
l. 328 Lefty1 and Mixl1 are mentioned, but I wasn't able to find the corresponding data.
l. 423: Gata6 and Eomes are mentioned as late genes. However, they are both already expressed at 48hpa (and even at 24hpa supp. fig.5). So, it appears difficult to relate this pseudotime with actual temporal events. Could it be that this pseudo late expression rather reflects and expression in cells further in the differentiation path? Just to be sure, since in figure d, the analysis is done specifically in the 24-48hpa, do the insets of Fig3a correspond to all integrated timepoints or just the 72hpa?
Similarly, l. 548-550: what do the authors mean by "associated with rising T expression"? Their reference to Turner et al. 2017 would suggest that they consider this relation to be causal. However, their pseudotime dynamic seems to occur later than T, thus according to these it would rather seem like secondary to T expression. I believe this reflect that it is risky to draw conclusion from pseudo-time analysis when temporal events are actually present in the data.
Alternatively, maybe using the latent time provided by the scvelo analysis might be more reflective of actual fate transition and better document their result.
Since this is not a major result of the manuscript it is up the authors to decide what they think of this comment.
Related to this single cell analysis, I am also unsure about the necessity of data integration in this context, it would in my opinion make more sense to just use these integrations either when comparing different single cell technologies or biological context with a large batch effect. Again, it is up to the authors to choose what they want to do regarding this comment.

Second revision

Author response to reviewers' comments

We thank the reviewers for the feedback and will answer the individual points in detail below. We have also marked the major changes in red in the revised manuscript for the ease of following changes. Due to the limitations on the word count, as pointed out by the editorial team, we have had to shorten the text in the *Introduction* and *Discussion* sections.

Reviewer 1

Advance Summary and Potential Significance to Field:
For a summary of the contents of the paper see my review from the initial submission. The main change compared to the original submission is the removal of one main figure and the corresponding text describing the effects of timed signaling perturbations on gastruloid development. While these experiments gave very intriguing phenotypes, the authors were unable to come up with a mechanistic explanation, and to meaningfully link these observations with the other data in the original version of the manuscript. In the interest of flow and readability, I agree with the author's decision to remove this section. In addition, the authors have made a number of smaller changes, mostly through revisions to the text, but also through addition of new supplementary figures, where they quantify the motility of T/Bra+ cells, compare gene expression in pluripotency

© 2024. Published by The Company of Biologists under the terms of the Creative Commons Attribution License (https://creativecommons.org/licenses/by/4.0/).

between their own line and a published dataset, and showcase expression patterns of marker genes in UMAP space.

With the changes made in the revision, the manuscript is now much more readable and focused on an important question – the emergence of cellular diversity during early gastruloid development. By highlighting the importance of initial culture conditions, it nicely complements other recent studies on the same topic, and is therefore suitable for publication in Development.

We thank the reviewer for the positive evaluation of our efforts at addressing the reviews in the last round.

Reviewer 1 Comments for the Author:
Minor

Most of my points have been appropriately addressed. In a final version, the authors may want to consider two minor additions:

1. The quantification of the motility of T/Bra+ cells in the new Supp. Fig. 1i is nice. Can the authors test whether the motility of T/Bra-negative cells is different?

We agree that this would be a nice test to do and we have a manuscript in preparation where we analyse these aspects in detail. However, with the cytoplasmic label we have used for this paper, the T/Bra- cells are unlabelled and therefore impossible to track. We tried labelling cells with nuclear dye (sir-DNA) but the stains were not robust to allow tracking of unlabelled cells. In light of these limitations, we have ensured that our conclusions in section 4.2 (and specially lines 251-253 that refer to Figure S1i) do not rely on comparison of T+ and T- cells but only on the observation of the *rich diversity in behaviours of T+ cells*. We hope this cautionary approach in the text is acceptable.

2. I am still missing an explanation (in the methods section) how an axis was determined in gastruloids at 24h and 48h that are still mostly round (minor point 6. from the initial round of reviews).

We agree that this was not very clear and apologise for the oversight on our part in addressing this. We have now added a new paragraph (lines 1034 - 1045 in the Methods section *6.3 Widefield imaging and quantitative analysis of gastruloids*) explaining this in detail. In a nutshell, we agree that the midline determination (and thus the “AP”-direction) from the (near-spherical) mask of an individual gastruloid at early stages (specially 24hpa) can be arbitrary. When averaged across several replicates in an experimental batch, this is indeed reflected in the near-flat profile of mean T-intensity along the “AP”-axis (Fig 1A, 1C). However, in batches where we do see beginning of polarisation, the mask tends to capture the concomitant small changes in shape and thus the AP-axis determination becomes more robust.

Reviewer 2

Advance Summary and Potential Significance to Field:
Anlaş, Gritti and colleagues provided here a well-performed study on gastruloids, a topical in vitro model of embryonic development where they show that lineage specification and patterning can occur prior (and in absence) of external cues (CHIR). The authors have done a commendable job in improving their manuscript and its readability. I am now in support of going forwards with a publication.

We appreciate the support of the reviewer and are very grateful for the positive assessment.

Reviewer 2 Comments for the Author:
The authors make a correct assessment that rearranging the text in a major fashion would constitute a major revision, and I think removing the signaling perturbations made the manuscript much more fluid so the option A which is the current one sufficiently conveys their message.
I think the authors have done a tremendous job improving their manuscript and its readability, the comparison with the early mouse single cell dataset is compelling although, it is surprising to see so little overlap since other datasets seem to show

© 2024. Published by The Company of Biologists under the terms of the Creative Commons Attribution License (https://creativecommons.org/licenses/by/4.0/).

better correspondence with mouse epiblast (Suppinger et al 2023), maybe this is due to the ESC culture conditions.
The point made by the authors regarding media composition from seemingly similar denotation (ESL) while using different actual media GMEM, DMEM +KO or KO DMEM is an important one, and may very well explain the different behavior observed between the line used in this study and the E14tg2a shown in Fig. supp. 2. Good point.

Once again, we are thankful.

Minor comments:
Supp. Fig. 1e isn't cited

We have fixed it in line 196.

l. 151 and Figure 1c: the authors state a limited overlap between T and Sox2 signal, however it seems that the Sox2 signal (in the quantification) is quite homogeneous at that stage while T already shows the initiation of polarization. In contrast, as stated l. 152-155, the 48hpa quantification is clearly showing spatial segregation of the two. Maybe one could phrase line 151 as: at 24hpa, while T signal already show polarization, Sox2 signal appears more homogeneous throughout gastruloids.

We agree and have rephrased this in lines 181-184 as "*At 24hpa,in relation to T signal which may already show initial polarization, Sox2 appears more homogeneous throughout gastruloids (Fig. 1C).*"

l. 164 typo where two "." are present.

This has been fixed.

l. 328 Lefty1 and Mixl1 are mentioned, but I wasn't able to find the corresponding data.

This has been corrected now in lines 367-370.

l. 423: Gata6 and Eomes are mentioned as late genes. However, they are both already expressed at 48hpa (and even at 24hpa supp. fig.5). So, it appears difficult to relate this pseudotime with actual temporal events. Could it be that this pseudo late expression rather reflects and expression in cells further in the differentiation path? Just to be sure, since in figure d, the analysis is done specifically in the 24-48hpa, do the insets of Fig3a correspond to all integrated timepoints or just the 72hpa?

We thank the reviewer for pointing this out and have now revised the text (lines 464-477) to include a word of caution about the differences that exist between pseudotime and actual temporal events. The insets correspond to the integrated dataset and we have now stated this in the figure legend.

Similarly, l. 548-550: what do the authors mean by "associated with rising T expression"? Their reference to Turner et al. 2017 would suggest that they consider this relation to be causal. However, their pseudotime dynamic seems to occur later than T, thus according to these it would rather seem like secondary to T expression. I believe this reflect that it is risky to draw conclusion from pseudo-time analysis when temporal events are actually present in the data.
Alternatively, maybe using the latent time provided by the scvelo analysis might be more reflective of actual fate transition and better document their result.
Since this is not a major result of the manuscript it is up the authors to decide what they think of this comment.

The reviewer has made a very important suggestion and we agree. Therefore, we have shortened the text (lines 601-606) to remove any implications of causality and rephrased it to explicitly state that the peaks observed are along the **pseudotime axis**.

Related to this single cell analysis, I am also unsure about the necessity of data integration in this context, it would in my opinion make more sense to just use these integrations either when comparing different single cell technologies, or biological context with a large batch effect. Again, it is up to the authors to choose what they want to do regarding this comment.

We appreciate this comment and would like to explain our rationale for doing data integration. As it is known, due to the inherent nature of the technique there are always

© 2024. Published by The Company of Biologists under the terms of the Creative Commons Attribution License (https://creativecommons.org/licenses/by/4.0/).

batch-effects, even when comparing similar/the same biological samples across timepoints. Therefore, to achieve reliable clustering of similar cells across timepoints, the field has employed several strategies to achieve batch correction, e.g. via data integration algorithms (Stuart *et al.*, Cell 2019, Seurat v3 in our case), or removal of those variable genes known to contribute to batch effects (Mayran *et al.*, bioRxiv 2023). Out of these practices we opted for data integration for our samples.

Third decision letter

MS ID#: DEVELOP/2023/202171

MS TITLE: Early, autonomous patterning of the anteroposterior axis in gastruloids

AUTHORS: Kerim Anlas, Nicola Gritti, Fumio Nakaki, Laura Salamo Palau, Sham Leilah Tlili, David Oriola, Krisztina Arato, Jia Le Lim, James Sharpe, and Vikas Trivedi

I am happy to tell you that your manuscript has been accepted for publication in Development, pending our standard publication integrity checks.

Reviewer 1

Advance summary and potential significance to field

Anlas, Gritti and colleagues present a single cell characterisation of the early stages of cell type diversification in gastruloids starting from cells maintained in serum + LIF conditions.
They report a surprisingly early expression of lineage markers and concomitant polarisation of the gastruloids, even before the Chiron pulse commonly applied in standard gastruloid formation protocols. They also demonstrate robustness of gastruloid development to the number of starting cells, and - by mapping their single cell data to reference atlases from the mouse embryo - propose that cells in gastruloids start from a developmental state that differs from that present in the embryo, but then converge to more similar cell identities. This study constitutes an important reference for mapping the mechanisms underlying self-organised cell differentiation and patterning in early mammalian development with stem cell models.

Comments for the author

The authors have appropriately addressed all comments from the previous rounds of review. I now fully endorse publication of the manuscript in its current form.

One minor amendment that the authors might want to make in the final version is a clearer description (e.g. in the caption of Fig. S8) of the differences in the dataset integration between Fig. 6A, B, and Fig. S8 A, B. It is not easy to decode from the captions as they stand why the transcriptomes from the present work map to different parts of the same reference in the two figures.

Lines 782/783: Typo, two times “core”

Reviewer 2

Advance summary and potential significance to field

I have no further comments, I believe the manuscript is ready for publication

Comments for the author

© 2024. Published by The Company of Biologists under the terms of the Creative Commons Attribution License (https://creativecommons.org/licenses/by/4.0/).

I thank the authors for the great improvements they have done on their manuscript which I believe is now ready for publication.

© 2024. Published by The Company of Biologists under the terms of the Creative Commons Attribution License (https://creativecommons.org/licenses/by/4.0/).
